# Metagenomic insights into microbial community alterations and co-occurrence networks in infective endocarditis

**DOI:** 10.1186/s44342-025-00059-y

**Published:** 2025-12-02

**Authors:** Zahra Abedi, Mohammad Ali Sheikh Beig Goharrizi, Amirreza Abbasi, Negar Sadat Soleimani Zakeri, Helia Jangi

**Affiliations:** 1 Dina Pharmed Exir Salamat, Pharmaceutical Co., Tehran, 574768 Iran; 2 Department of Medical Biotechnology, School of Biotechnology, College of Science, University of Tehran, Tehran, Iran; 3 Department of Health Science, University of Eastern Piedmont, Novara, Italy; 4 Department of Software Engineering, Engineering and Architecture Faculty, Istanbul Nişantaşi University, Istanbul, Turkey; 5 Department of Science and Technological Innovation, University of Eastern Piedmont, Novara, Italy

**Keywords:** Computational analyses, Infective endocarditis, Metagenome, Microbiome, 16S rRNA

## Abstract

**Background:**

Infective endocarditis (IE) is a serious infection of the heart valves, and standard culture methods often miss the bacteria responsible, especially in culture-negative cases. To address this, we used 16S rRNA gene-based next-generation sequencing (NGS) on heart valve tissue. This approach allowed us to map out the bacterial communities present and evaluate their potential role in IE.

**Result:**

We identified six key bacterial genera—*Enterococcus*, *Streptococcus*, *Coxiella*, *Staphylococcus*, *Haemophilus*, and *Cutibacterium—plus three specific* species: *Streptococcus troglodytae*, *Haemophilus parainfluenzae*, and *Coxiella burnetii*. Our co-occurrence analysis showed that these bacteria tend to exist independently within infected valve tissue, with no significant correlations between them.

**Conclusion:**

We detected bacterial taxa, including *Cutibacterium* and *Streptococcus troglodytae*. Although *S. troglodytae* is rarely associated with IE, and *Cutibacterium* comprises low-abundance bacteria not typically linked to this condition. These findings demonstrate the value of NGS in identifying pathogens that standard culture methods may overlook. As these results are based on computational analyses, further laboratory validation is required. Incorporating NGS into diagnostic protocols may enhance pathogen detection in culture-negative IE and support more targeted treatment and prevention strategies.

**Supplementary Information:**

The online version contains supplementary material available at 10.1186/s44342-025-00059-y.

## Introduction

Infective endocarditis (IE) is a rare but life-threatening inflammation affecting the heart’s inner lining (endocardium), valves, atria, and ventricular walls. It can appear in three forms: (a) acute—symptoms develop within days and last up to 6 months, (b) subacute—symptoms appear over 6 weeks to 3 months, and (c) chronic—symptoms persist for more than 3 months. Although uncommon, IE ranks as the third or fourth most severe infectious syndrome, with in-hospital mortality of 20–25% and an annual incidence of 3–10 cases per 100,000 people [1].

The disease is caused by bacteria, fungi, or viruses entering the bloodstream, with *Streptococcus* and *Staphylococcus* species responsible for about 80% of cases. *Staphylococcus aureus* is particularly aggressive, especially in older or vulnerable patients [2]. Blood culture (BC) testing remains the diagnostic gold standard, yet 2.5–31% of IE cases are BC-negative. In suspected IE, additional microbiological, histopathological, serological, and PCR tests are recommended [3, 4]. Challenges such as false negatives and slow results make early detection and prevention essential [5].

Molecular techniques like next-generation sequencing (NGS) can help overcome the limits of culture-based methods and BC false negatives [6, 7]. These technologies, paired with computational analysis, allow the identification of causative pathogens by examining the microbiome of specific organs, including the heart [8]. The microbiome—defined as the complete set of microbial communities in the body—plays a role in health and disease [9]. The Human Microbiome Project identifies about 48 microbial habitats in the human body, with interactions affecting inflammatory and metabolic pathways [10]. Disruptions in microbial balance, known as dysbiosis, are linked to diseases involving the immune and metabolic systems, such as diabetes and obesity [11, 12].

In IE, the main pathogens remain *staphylococci* (31% of cases), *streptococci*, and *enterococci*, with less common agents like *Candida* and *Pseudomonas aeruginosa *[13]. Infection typically begins when pathogens attach to damaged heart valves, binding to surfaces coated with fibronectin, plasma proteins, platelet proteins, and fibrin [14]. This leads to colonization, thrombosis, inflammation, and vegetation formation [15]. Research increasingly suggests that understanding the specific functions of these microbes—rather than just changes in their abundance—offers deeper insight into disease mechanisms [16, 17].

Transient bacteremia, often caused by injury to mucosal or skin surfaces rich in native microflora, is an important risk factor for IE [18]. The disease is also associated with periodontitis, a chronic gum infection caused by specific oral bacteria [19]. Similar to findings in gut microbiome studies, bioactive metabolites produced by microbes can influence cardiovascular and metabolic health [20]. Comparing microbiota from healthy individuals with those from IE patients can help identify functionally relevant species, potentially aiding in prevention and treatment [21, 22].

Co-occurrence pattern analysis is another valuable approach. By examining how the species tend to appear together, researchers can uncover ecological relationships, shared niches, and potential interactions that help to maintain the microbial diversity or drive the disease [23, 24]. This method is particularly useful when a microorganism’s biology is poorly understood, as it can reveal functional roles and interaction networks [25].

Our study applied 16S rRNA gene-targeted NGS to heart tissue from IE patients [26]. Using the QIIME2 bioinformatics pipeline, we identified microbial taxa at the family, genus, and species levels and investigated diversity and co-occurrence patterns to gain insights into the microbial ecology of infective endocarditis [27–29].

## Material and methods

### Study design, dataset, and bioinformatics analysis

The accession number of data that was used in this study was PRJNA701379.

Data from the PRJNA701379 BioProject, initially reported by Santibanez et al. (Pathogens, 2021) [29], were reanalyzed to assess reproducibility and apply updated bioinformatics and statistical methods. The dataset included 27 heart valve tissue samples from patients with infective endocarditis (IE): 4 prosthetic valves, 5 intravascular devices, and 18 native valves, with four culture-negative cases. All analyses used de-identified, publicly available data, so ethical approval was not required [1]. Each sample contained 16S rRNA extracted from heart valve tissue.

**Fig. 1:**
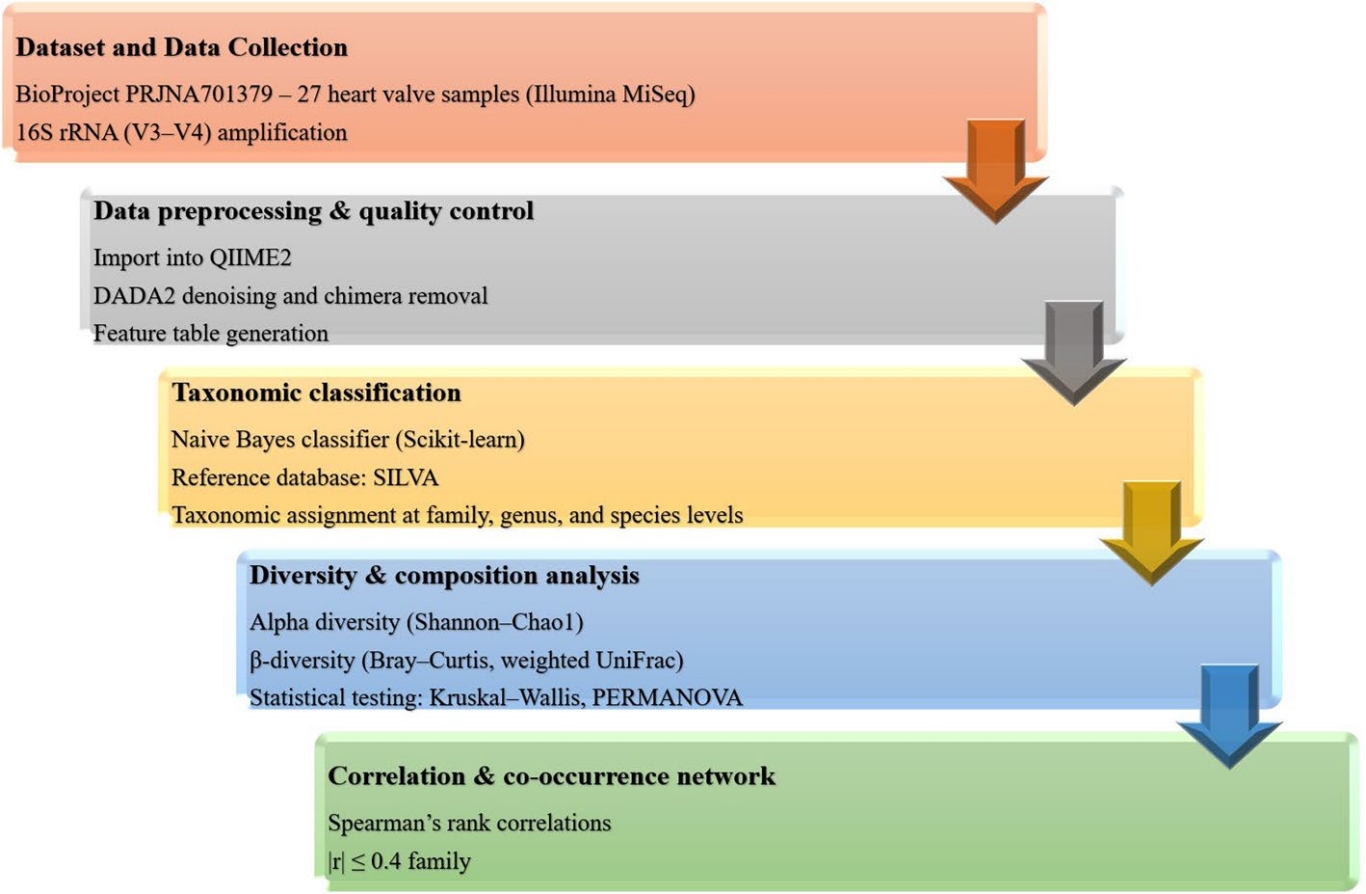
Schematic of bioinformatics workflow and statistical analyses

Data were downloaded from the EBI database (https://www.ebi.ac.uk/) (accessed 26 September 2025), and metadata were saved as text files. Additional details are provided in Table 1. Raw data were processed using the same sequencing platform and methods as the original study, which used paired-end reads from the Illumina platform. Two Fastq files, one for forward reads and one for reverse reads, were generated. These files were demultiplexed using the Quantitative Insights into Microbial Ecology (QIIME2 version 2024.10.1) [24] (https://qiime2.org/) platform. This step produced a summary of sequence quality and sequence counts per sample. All bioinformatics analyses were performed using the QIIME2 platform (version 2024.10.1) with standard pipelines, and all parameters were reported to ensure reproducibility and transparency. Figure 1 presents a schematic workflow of the analysis.

**Table 1:** Details of the used data in this study

Disease	Infective endocarditis
Data type	16SrRNA
Platform	Illumina
Instrument	MiSeq
Sequencing type	Paired
Accession	PRJNA701379
16SrRNA region sequencing	V3-V4
Patient	27
Tissue type	Heart valve tissue
Country	Spain

### Denoising data

We used the DADA2 [30] plugin in QIIME2 (version 2024.10) to check sequence quality and remove noise. This step corrects Illumina sequencing errors, removes chimeric reads, and identifies amplicon sequence variants (ASVs) at single-nucleotide resolution. We set truncation lengths to 240 bp for forward reads and 200 bp for reverse reads, based on the quality profiles of the raw data. This provided about 20 bp of overlap for merging paired-end reads and helped avoid including low-quality bases at the 3′ ends. After denoising and removing chimeric and singleton reads, we set the rarefaction depth for diversity analyses to 500. We chose this value based on the sequence count distribution to keep most samples and ensure enough coverage. To reduce the effect of contaminants from reagents or the environment, we excluded low-abundance and unclassified features identified as potential contaminants using prevalence patterns and previous studies before further analysis.

### Taxonomic classification

Taxonomic classification of representative sequences was conducted in QIIME2 using a Naive Bayes classifier (Scikit-learn implementation) [31]. Reference sequences and taxonomy were sourced from the SILVA 138 SSURef NR99 database (https://www.arb-silva.de) [32], which offers comprehensive, current, and accurate annotation of bacterial 16S rRNA sequences. SILVA RNA sequences were reverse transcribed to DNA, cleaned, and dereplicated before classifier training. The V3–V4 region (341F/806R primers) was extracted from reference sequences to match our dataset amplicons. The trained classifier assigned taxonomy to all representative sequences at the phylum, class, genus, and species levels. Sequences identified as mitochondria, chloroplast, or non-bacterial/uncultured taxa were removed before further analysis. Taxonomic assignments were then tabulated and visualized.

### Alpha diversity measurement

To measure phylogenetically based α-diversity, we built a phylogenetic tree using a fast tree program and rooted it at the midpoint. Since the tree was constructed from a single marker gene, we avoided making strong conclusions about taxonomic relationships. After generating α- and β-diversity data, we calculated α-diversity indices such as Shannon and Observed ASVs, using a rarefaction depth of 500 sequences per sample to standardize sampling effort. We tested relationships between α-diversity metrics and metadata categories with the Kruskal–Wallis test.

We visualized β-diversity metrics, including weighted and unweighted UniFrac and Bray–Curtis distances, with principal coordinates analysis (PCoA). We used PERMANOVA to assess differences between sample groups.

### Co-occurrence analysis

Co-occurrence patterns among bacterial taxa were evaluated using Spearman’s rank correlation in R (v. 4.0.4). Both positive (co-occurrence) and negative (mutual exclusion) associations were analyzed. The analysis was conducted at the family and genus levels using relative abundance matrices generated from QIIME2 outputs. Correlations with absolute values of |*r*|≥ 0.4 at the family level and |*r*|≥ 0.6 at the genus level, with *p* < 0.1, were selected for network construction. To reduce spurious associations, taxa with low prevalence (fewer than two samples) or ambiguous taxonomic assignments were excluded prior to analysis. The resulting correlation matrices were prepared for potential evaluation. However, no robust co-occurrence network could be established under these thresholds.

## Result

### Dataset and preprocessing

The metadata for 16S rRNA with accession number PRJNA701379 was utilized in this study [1] and presents additional details regarding these data. QIIME2 was employed for data analysis. The dataset comprised 27 paired-end samples with a total frequency of 23,988 prior to filtering. The summary in Table 1 indicated a total frequency of 23,988, 774 features, and 27 samples. The minimum and maximum frequencies were 0 and 5698, respectively. Additional information is available in Supplementary Tables S1 and S2. Feature counts and detailed feature information for each sample are presented in Supplementary Tables S3 and S4, respectively. Quality control was performed as an essential preprocessing step for raw sequencing reads prior to further analysis. Demultiplexing and quality filtering of the sequences were conducted. The summary of demultiplexed sequence counts is shown in Supplementary Table S5. According to Supplementary Fig. S1, the total frequency in demultiplexed sequence counts was 11,599,552. Sequence counts per sample are illustrated in Supplementary Table S6. Quality plots are provided in Supplementary Figs. S2 and S3. Supplementary Table S7 summarizes the total sequences sampled in forward and reverse reads, as well as the lengths of demultiplexed sequences. Following demultiplexing, quality control, and contaminant filtering, the final dataset consisted of 25 samples, 650 features, and 22,851 total sequences (see Supplementary Tables S8 and S9 for filter table summaries). The sequence frequency per sample ranged from 2 to 5166, with a median of 621 and a mean of 914. Based on these results, a rarefaction depth of 500 sequences per sample was selected for diversity analyses. Further details are available in Supplementary Tables S8 and S9.

### Taxonomic composition and taxa bar plots

**Fig. 2:**
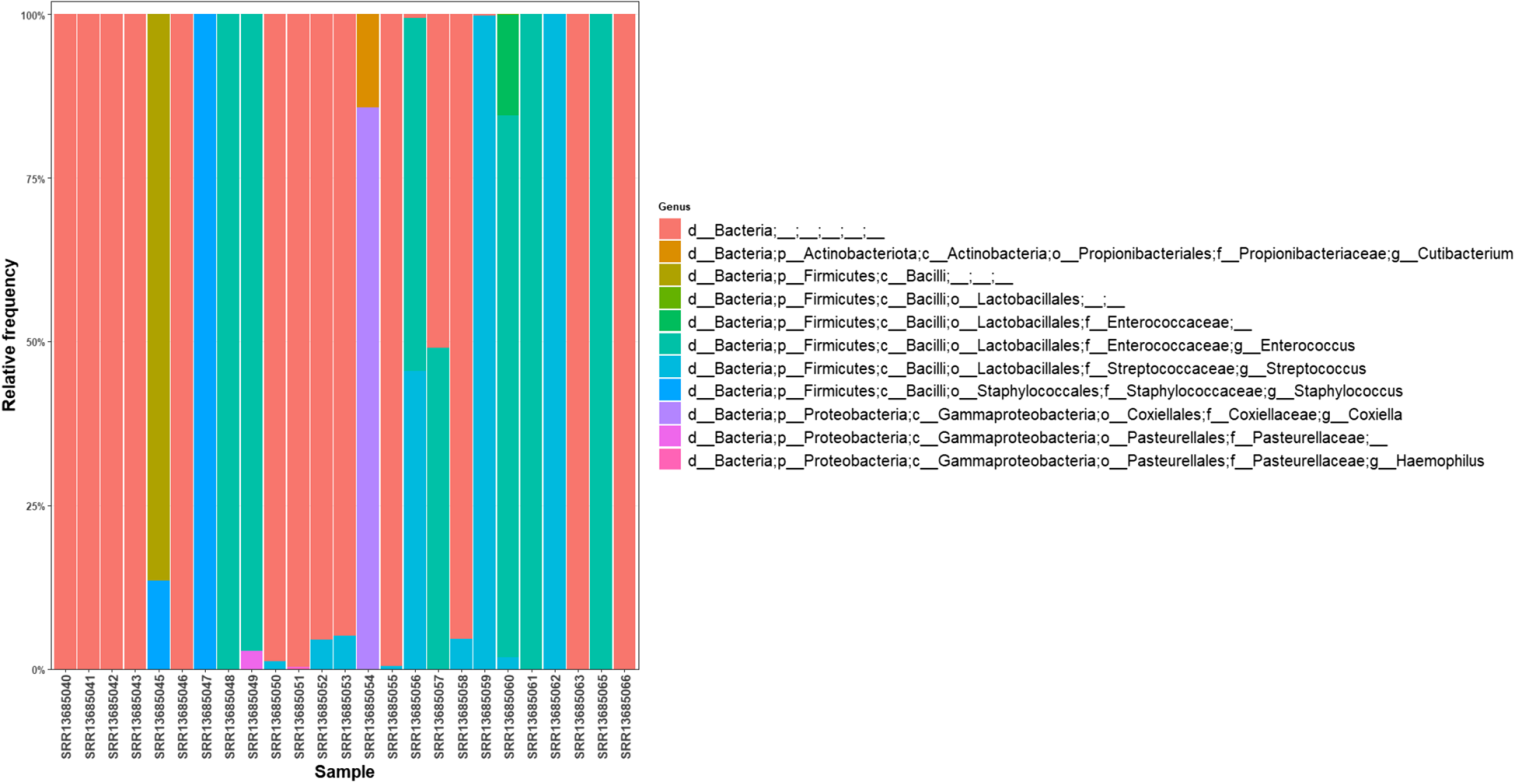
Taxa bar plot at level the genus level (level 6). Dominant genera include *Enterococcus*, *Streptococcus*, *Coxiella*, *Staphylococcus*, *Haemophilus*, and *Cutibacterium*

**Fig. 3:**
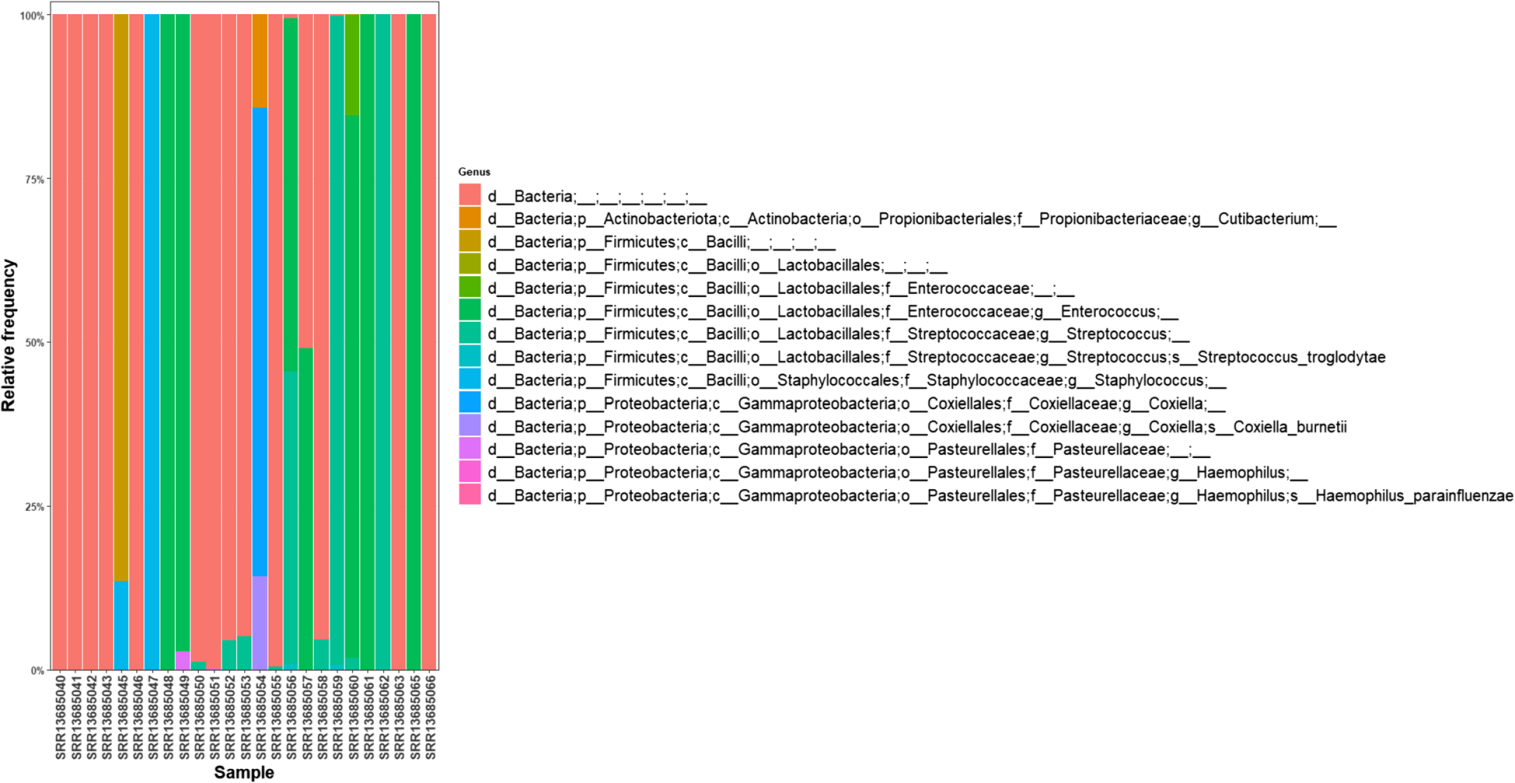
Taxa bar plot at the species level (level 7). Three species were surely classified: *Streptococcus troglodytae*, *Haemophilus parainfluenzae*, and *Coxiella burnetii*

We analyzed the taxonomic composition of infective endocarditis samples using the QIIME2 Naive Bayes classifier, trained on the SILVA 138 database. This approach allowed us to identify the main bacterial groups present in each sample with a high level of confidence. After filtering and rarefaction, we retained 25 samples for further analysis. The taxonomic assignments were reliable up to the genus level, but as expected with 16S rRNA gene sequencing, species-level resolution was limited. This is a known limitation of the method, so results at the genus level are the most actionable. Our genus-level analysis (see Fig. 2) showed that six bacterial genera dominated the samples: *Enterococcus*, *Streptococcus*, *Coxiella*, *Staphylococcus*, *Haemophilus*, and *Cutibacterium*. At the species level, we could confidently identify three key taxa: *Streptococcus troglodytae*, *Haemophilus parainfluenzae*, and *Coxiella burnetii* (see Fig. 3). We found these taxa mainly in samples from both native and prosthetic valve tissues. This pattern points to possible microbial differences between clinical subgroups, which could be important for diagnosis and treatment planning. Importantly, we detected *Coxiella burnetii* and *Haemophilus parainfluenzae* in cases where traditional cultures were negative. This shows that metagenomic sequencing can add real diagnostic value in infective endocarditis, especially when standard methods fall short.

### Alpha and beta diversity indexes and curves rarefaction

#### Alpha diversity analysis

**Fig. 4:**
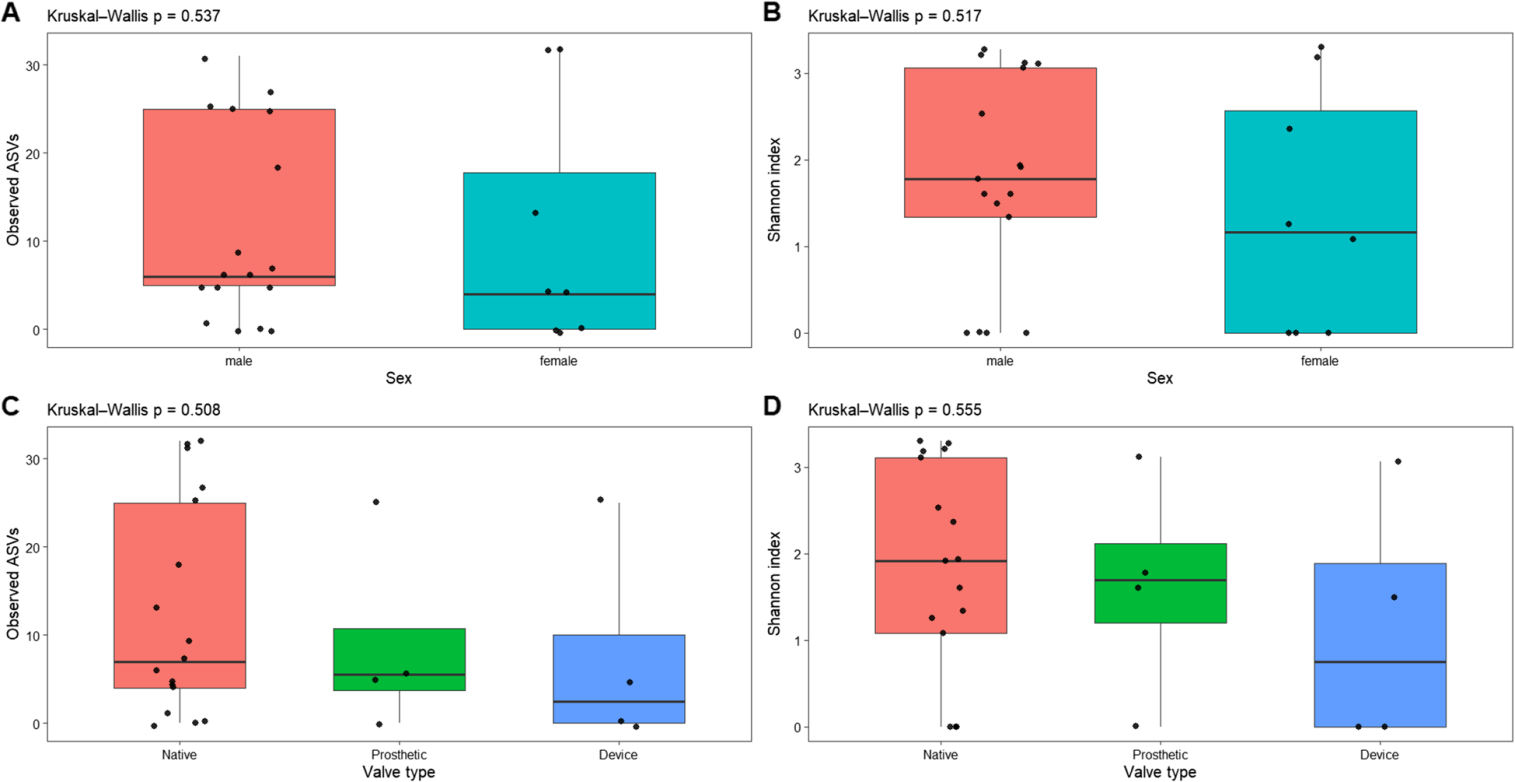
Alpha diversity of IE samples. **a** Shannon index by tissue type. **b** Observed ASVs by sex. No significant differences were detected (Kruskal–Wallis, *p* > 0.05)

Alpha diversity was evaluated using the Observed ASVs (microbial richness) and Shannon diversity (richness and evenness) indices to examine within-sample diversity in infective endocarditis samples. Observed ASVs ranged from 9 to 128, showing that microbial richness varied among patients. When grouped by sex, female samples had slightly higher median richness and diversity than male samples. However, these differences were not statistically significant for either index (observed ASVs*: p* = 0.537; Shannon:* p* = 0.517) (Fig. 4a, b). Grouping samples by tissue type—native and prosthetic valves, and intravascular devices—also showed no significant differences in alpha diversity (observed ASVs: *p* = 0.69; Shannon*: p* = 0.64) (Fig. 4c, d). Although these differences were not statistically significant, native valve samples, especially native mitral valves, tended to have higher microbial richness than prosthetic and device-associated samples. Rarefaction curves plateaued at the selected sequencing depth of 500 sequences per sample, indicating sufficient coverage for estimating within-sample diversity (Supplementary Fig. S1). In summary, alpha diversity results showed moderate differences in microbial richness and evenness across infective endocarditis samples, with no significant differences based on sex or tissue type.

#### Beta diversity analysis

**Fig. 5:**
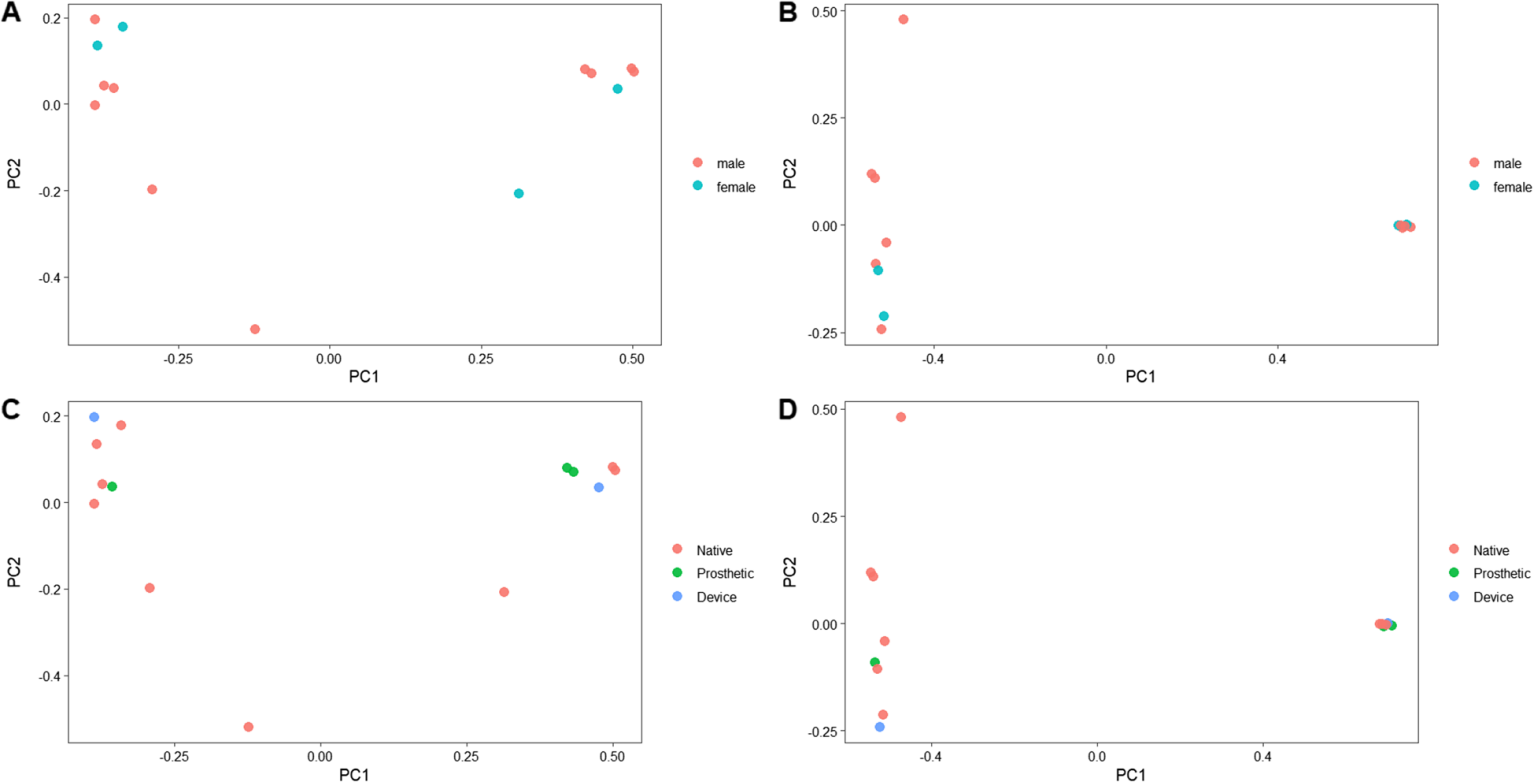
Beta diversity of infective endocarditis (IE) samples. Principal Coordinates Analysis (PCoA) plots based on unweighted and weighted UniFrac distances are shown. **A**–**B** Samples colored by sex (**A** = unweighted, **B** = weighted). **C**–**D** Samples colored by valve type (**C** = unweighted, **D** = weighted). The plots indicate substantial overlap and no distinct clustering among groups, indicating similar microbial community compositions across sex and valve types

**Fig. 6:**
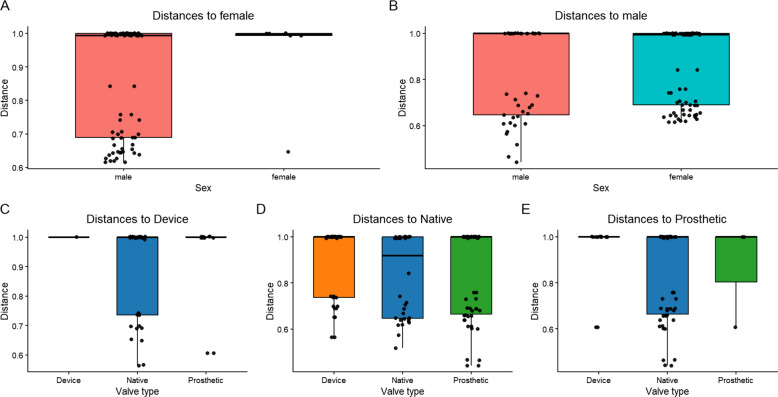
Pairwise beta diversity distances among infective endocarditis (IE) samples. Boxplots illustrate pairwise Bray–Curtis dissimilarities between sample groups. **A**–**B** Sex-based comparisons showing distances to female (**A**) and to male (**B**) samples. **C**–**E** Valve-type comparisons showing distances to Device (**C**), Native (**D**), and Prosthetic (**E**) valves. Boxplots indicate median, interquartile range, and individual pairwise distances; no significant differences were observed between groups (PERMANOVA, *p* > 0.05)

Beta diversity metrics were used to assess differences in microbial community structure among samples. Principal coordinates analysis (PCoA) using unweighted UniFrac distances showed that male and female samples overlapped a lot, suggesting their phylogenetic composition was similar regardless of sex (Fig. 5). Similarly, the weighted UniFrac PCoA, which incorporates relative abundances, demonstrated no distinct clustering between the two groups (Fig. 5). PERMANOVA analysis of Bray–Curtis distances further confirmed that overall microbial community composition did not differ significantly between male and female patients (pseudo-*F* = 0.918, *R*^2^ = 0.019, *p* = 0.347; Fig. 6). These results suggest that, based on our data, sex did not have a noticeable effect on the beta diversity of valve-associated microbial communities in infective endocarditis. Beta diversity analyses by valve type (native, prosthetic, and device-associated) also showed no clear clustering between groups. Both unweighted and weighted UniFrac PCoA plots indicated overlapping microbial communities among valve categories (Fig. 5). In accordance with these observations, PERMANOVA tests using Bray–Curtis and weighted UniFrac distances identified no statistically significant differences in microbial composition (Fig. 6). These results indicate that microbial community structures associated with infective endocarditis were broadly similar across the different valve types.

#### Co-occurrence analysis

We used Spearman’s rank correlation analysis to assess potential co-occurrence and exclusion patterns among bacterial taxa. Applying the predefined thresholds (|*r*|≥ 0.4 for families and |*r*|≥ 0.6 for genera; *p* < 0.1), we found no significant pairwise correlations. As a result, stable co-occurrence networks could not be constructed at either the family or genus level. The absence of strong associations suggests that microbial communities in infective endocarditis tissues likely consist of independently distributed taxa rather than closely interacting consortia. We evaluated both positive and negative correlations, but none exceeded the significance thresholds. Complete correlation matrices are available in Supplementary Tables S10 and S11.

## Discussion

This study used 16S rRNA sequencing to analyze the microbes found in heart valve tissue from patients with infective endocarditis (IE). The goal was to identify known and new pathogens and to look for possible patterns in how these microbes appear together [19, 30]. No important patterns were found in how different microbes appeared together. The study confirmed the presence of well-known IE bacteria and also found some species that are rarely linked to the disease. This suggests that more types of bacteria may cause IE than previously thought [33]. IE is a serious inflammatory disease that happens when bacteria get into the bloodstream and stick to the endocardium, heart valves, or blood vessels. IE affects 3 to 10 people out of every 100,000 each year and can have a 1-month death rate as high as 30%. Finding the disease early is very important, especially for patients with risk factors like heart murmurs, vasculitis, or embolic events [34]. Even with major improvements in medical and surgical care, IE is still one of the most serious inflammatory diseases [35, 36].

We started with 25 heart valve tissue samples and ran them through the QIIME2 pipeline. After quality control, we excluded two samples with low sequence reads, leaving us with 23 high-quality samples for analysis. The analysis identified six bacterial genera: *Enterococcus*, *Streptococcus*, *Coxiella*, *Staphylococcus*, *Haemophilus*, and *Cutibacterium*. Three species were also found: *Streptococcus troglodytae*, *Haemophilus parainfluenzae*, and *Coxiella burnetii*. The microbial composition was dominated by *Enterococcus*, *Streptococcus*, and *Coxiella*, which are taxa frequently reported in cases of infective endocarditis. Finding *Streptococcus troglodytae*, which has not been reported before in infective endocarditis, adds to the list of possible pathogens. Genomic data show that this species has virulence-associated genes [37], suggesting it could colonize human tissues under certain conditions. This bacterium was first found in the mouths of chimpanzees, but its high sequence similarity to human-associated streptococci suggests it may have been misidentified, rather than transferred from animals to humans. The genome of *S. troglodytae* is more than 96% similar in glucan-binding protein genes (gbpA–D) to *S. mutans*, a common oral pathogen that can stick to collagen and play a role in infective endocarditis. *S. troglodytae* does not have the cnm and cbm genes linked to collagen binding, but its gbp genes may still help it stick to and colonize heart tissue [37, 38].

It is important to note that while the healthy heart is a sterile environment, infective endocarditis disrupts this sterility through bacterial colonization and biofilm formation on valve surfaces [39]. The bacterial DNA found in this study came directly from infected valve tissue, matching what we expect from the disease process and not from outside contamination. We used strict contamination controls throughout all bioinformatics and analysis steps. Common reagent or environmental contaminants like Pseudomonas and Ralstonia were not detected. In addition, the taxonomic assignment of *S. troglodytae* was based on 16S rRNA similarity to reference sequences in the SILVA database; therefore, it may represent a closely related human-associated streptococcal strain rather than a true zoonotic organism. The low microbial diversity and dominance of a few taxa show that valve-associated microbiota in infective endocarditis are stable, opportunistic communities, not highly diverse consortia. While the microbiomes of many organs have been studied, the specific bacterial communities in heart tissue are still poorly understood. Reports based on metagenomic analysis are scarce and often involve only a handful of patients [40, 41]. This underlines why next-generation sequencing (NGS) is so valuable for identifying the full range of infectious agents [42].

Previous studies have also found that *Enterococcus*, *Streptococcus*, and *Coxiella* are the main bacteria found in infective endocarditis (IE) valve tissues, highlighting their key role in the disease. These taxa account for the majority of cases, with *Streptococcus* and *Enterococcus representing* primary organisms identified on infected valve tissues and being closely associated with the pathophysiology of vegetation and biofilm formation [43, 44]. Although *Staphylococcus aureus* is a major global cause, the predominance of *Enterococcus*, *Streptococcus*, and *Coxiella* in specific patient cohorts is a well-documented pattern that supports the clinical diagnosis of infective endocarditis. *Coxiella burnetii* is especially important in cases of culture-negative endocarditis and often needs molecular methods for accurate detection [43, 44]. Gram-negative bacteria and *Streptococcus* species made up over 80% of IE cases, matching trends seen in many years of epidemiological data [45]. *Streptococcus*, *Staphylococcus*, and *Enterococcus* together made up 50–70% of the pathogens found in blood cultures from IE patients [46].

Among specific species, *Streptococcus troglodytae*, though rarely identified, may be detected by molecular methods but often represents close human-associated relatives when found in human valve tissue. *Haemophilus parainfluenzae*, a member of the HACEK group, is a recognized cause of infective endocarditis. It is often linked to pre-existing cardiac lesions or oral and dental sources, and may be underdiagnosed due to culture challenges [43, 44, 47]. *Coxiella burnetii* is an important cause of blood culture-negative endocarditis and often requires molecular or serological tests for detection [43]. *Cutibacterium* is rare but has been found in some prosthetic valve endocarditis cases [48].

Our findings reinforce the connection between oral health and heart infections. Detection of *Staphylococcus* in heart tissue supports evidence that common oral and skin commensals can move and contribute to infective endocarditis [49]. Similarities between oral and cardiac microbiota suggest that oral dysbiosis may contribute to infective endocarditis Along with *Streptococcus* and *Staphylococcus*, *Haemophilus parainfluenzae*, a member of the HACEK group, *was also found.* This matches its known role as an opportunistic pathogen in subacute endocarditis [50, 51]. *Enterococcus* and *Coxiella burnetii* were also detected, both of which are well-recognized infective endocarditis pathogens [52, 53]. *Cutibacterium*, typically a skin commensal, has been increasingly reported in prosthetic valve infections. This suggests that low-abundance organisms may sometimes access cardiac tissue and contribute to persistent infection [48, 54].

Other genera found, including *Enterococcus* and *Coxiella*, are recognized IE pathogens. *C. burnetii* is a leading cause of blood culture-negative endocarditis [55]. Although no statistically significant co-occurrence patterns were observed, the dominance of *Staphylococcaceae* and *Streptococcaceae* suggests these taxa may influence infection dynamics through competitive or synergistic interactions within the infected tissue. The prevalence of *Streptococcaceae* in both oral and cardiac samples supports the link between oral and heart health. Many streptococci adhere strongly to oral surfaces, a trait that may facilitate their attachment to heart valves [56, 57]. Overall, these findings indicate that IE pathogenesis is mainly driven by a limited group of opportunistic taxa capable of colonizing and persisting in cardiac tissues, rather than by complex polymicrobial communities.

## Conclusion

Metagenomic tools like next-generation sequencing (NGS) enable comprehensive mapping of microbiota composition through the 16S rRNA analysis, facilitating the identification of disease-associated microbial imbalances and showing potential therapeutic interventions. Researchers analyzed heart valve tissue from patients with infective endocarditis (IE) and found six bacterial genera: *Enterococcus*, *Streptococcus*, *Coxiella*, *Staphylococcus*, *Haemophilus*, and *Cutibacterium.* They also identified three species: *Streptococcus troglodytae*, *Haemophilus parainfluenzae*, and *Coxiella burnetii.* The finding that *Enterococcus*, *Streptococcus*, and *Coxiella* are most common matches earlier studies, which also identify these bacteria as main causes of infective endocarditis. The identification of *S. troglodytae*, a species previously isolated from the oral cavity of chimpanzees and closely related to human-associated streptococci, broadens the recognized spectrum of potential IE pathogens. Because *S. troglodytae* is genetically similar to *S. mutans,* especially in glucan-binding protein genes (*gbpA–D*), related oral bacteria may have adhesive traits that help them stick to heart tissue. *Haemophilus parainfluenzae*, a member of the HACEK group, and *Cutibacterium* were also identified, supporting the contribution of both oral and commensal microbiota to valve infection. There was no significant co-occurrence patterns among the bacteria found. This suggests that infective endocarditis is usually caused by a few opportunistic bacteria that can stick to and survive in heart tissue, rather than by complex groups of many microbes. Overall, these findings highlight the value of next-generation sequencing (NGS) for detecting both common and rare pathogens, particularly in culture-negative IE cases. Adding NGS to diagnostic routines can help identify pathogens more accurately, clear up unclear clinical cases, and allow for more precise treatment of infective endocarditis. Although more clinical testing is needed, using NGS regularly could improve early detection and support personalized treatment for this condition.

## Supplementary Information

Supplementary Material 1: Table S1. Table summary before filtering. Table S2. Details of frequency based on frequency in per sample. Table S3. Feature counts in each sample. Table S4. Feature details in each sample. Table S5. Demultiplexed sequence counts summary. Table S6. Sequence counts in per sample. Table S7. Demultiplexed sequence length summary. Table S8. Table summary after filtering. Table S9. Frequency per sample after filtering. Table S10. Kruskal–Wallis results for alpha diversity. Table S10. The correlations between identified families based on Spearman's correlation. Table S11. The correlations between identified genera based on Spearman's correlation. Figure S1. Demultiplexed sequence counts summary plot (A) Forward Reads Frequency, (B) Reverse Reads Frequency. Figure S2. Quality plot for forward reads (These plots were generated using a random sampling of 10000 out of 11599552 sequences without replacement. The minimum sequence length identified during subsampling was 300 bases. Outlier quality scores are not shown in box plots for clarity.) Figure S3. Quality plot for reverse reads (The plot at position 131 was generated using a random sampling of 10000 out of 11599552 sequences without replacement. The minimum sequence length identified during subsampling was 300 bases. Outlier quality scores are not shown in box plots for clarity)

## Data Availability

Availability of data and materials:  The datasets utilized in this study can be provided by the corresponding author on reasonable request. The raw dataset is available on European Bioinformatics Institute (EBI) with PRJNA701379 accession number. (https://www.ebi.ac.uk/ena/browser/view/PRJNA701379?show=reads).

## References

[1] Liu F, Fan C, Zhang L, Li Y, Hou H, Ma Y, et al. Alterations of gut microbiome in Tibetan patients with coronary heart disease. Front Cell Infect Microbiol. 2020;10:373. 32793515 10.3389/fcimb.2020.00373 PMC7390946

[2] Paul G, Ochs L, Hohmann C, Baldus S, Michels G, Meyer-Schwickerath C, et al. Surgical procedure time and mortality in patients with infective endocarditis caused by *Staphylococcus aureus* or *streptococcus* species. J Clin Med. 2022;11(9):2538. 35566663 10.3390/jcm11092538 PMC9104614

[3] Tran MP, Caldwell-McMillan M, Khalife W, Young VB. *Streptococcus intermedius* causing infective endocarditis and abscesses: a report of three cases and review of the literature. BMC Infect Dis. 2008;8(1):154. 18992173 10.1186/1471-2334-8-154 PMC2600825

[4] Janda WM. Update on family Pasteurellaceae and the status of genus *Pasteurella* and genus *Actinobacillus*. Clin Microbiol Newsl. 2011;33(18):135–44.

[5] Saraithong P, Pattanaporn K, Chen Z, Khongkhunthian S, Laohapensang P, Chhun N, et al. *Streptococcus mutans* and *Streptococcus sobrinus* colonization and caries experience in 3-and 5-year-old Thai children. Clin Oral Invest. 2015;19(8):1955–64. 10.1007/s00784-015-1437-0 PMC4886470 25753978

[6] Nishimura S, Matsuyama S, Yamamoto K. *Staphylococcus saprophyticus* native valve endocarditis possibly originating from the lower gastrointestinal tract. IDCases. 2020;19:e00713. 32099810 10.1016/j.idcr.2020.e00713 PMC7031001

[7] Barberán A, Bates ST, Casamayor EO, Fierer N. Using network analysis to explore co-occurrence patterns in soil microbial communities. ISME J. 2012;6(2):343–51. 21900968 10.1038/ismej.2011.119 PMC3260507

[8] Salgado-Pabón W, Breshears L, Spaulding AR, Merriman JA, Stach CS, Horswill AR, et al. Superantigens are critical for *Staphylococcus aureus* infective endocarditis, sepsis, and acute kidney injury. MBio. 2013;4(4):e00494-13. 23963178 10.1128/mBio.00494-13 PMC3747586

[9] Bartelli TF, Vaz Coelho LG, Dias-Neto E. Chapter 10 - The gastric microbiome in benign and malignant diseases. In: Faintuch J, Faintuch S, editors. Microbiome and Metabolome in Diagnosis. Therapy, and other Strategic Applications: Academic Press; 2019. p. 101–8.

[10] Otto M. Staphylococci in the human microbiome: the role of host and interbacterial interactions. Curr Opin Microbiol. 2020;53:71–7. 32298967 10.1016/j.mib.2020.03.003

[11] Cahill TJ, Baddour LM, Habib G, Hoen B, Salaun E, Pettersson GB, et al. Challenges in infective endocarditis. J Am Coll Cardiol. 2017;69(3):325–44. 28104075 10.1016/j.jacc.2016.10.066

[12] Miro JM, Anguera I, Cabell CH, Chen AY, Stafford JA, Corey GR, et al. *Staphylococcus aureus* native valve infective endocarditis: report of 566 episodes from the International Collaboration on Endocarditis Merged Database. Clin Infect Dis. 2005;41(4):507–14. 16028160 10.1086/431979

[13] Fujita H, Nakamura I, Tsukimori A, Sato A, Ohkusu K, Matsumoto T. Severe infective endocarditis in a healthy adult due to *Streptococcus agalactiae*. Int J Infect Dis. 2015;38:43–5. 26188131 10.1016/j.ijid.2015.07.009

[14] Lamas CdC, Ramos RG, Lopes GQ, Santos MS, Golebiovski WF, Weksler C, et al. *Bartonella* and *Coxiella* infective endocarditis in Brazil: molecular evidence from excised valves from a cardiac surgery referral center in Rio de Janeiro, Brazil, 1998 to 2009. Int J Infect Dis. 2013;17(1):e65–6. 23219032 10.1016/j.ijid.2012.10.009

[15] Lu H, Zhao W, Liu WH, Sun T, Lou H, Wei T, et al. Safety evaluation of *Bifidobacterium lactis* BL-99 and *Lacticaseibacillus paracasei* K56 and ET-22 *in vitro* and *in vivo*. Front Microbiol. 2021;12:686541. 34394030 10.3389/fmicb.2021.686541 PMC8358461

[16] Magarifuchi H, Kusaba K, Yamakuchi H, Hamada Y, Urakami T, Aoki Y. *Staphylococcus saprophyticus* native valve endocarditis in a diabetic patient with neurogenic bladder: a case report. J Infect Chemother. 2015;21(9):695–9. 26184852 10.1016/j.jiac.2015.05.008

[17] Hogevik H, Söderquist B, TUNG HS, Olaison L, Westberg A, Rydén C, et al. Virulence factors of Staphylococcus aureus strains causing infective endocarditis–a comparison with strains from skin infections. APMIS. 1998;106(7‐12):901–8. 9808417

[18] Saarela M, Matto J, Mattila-Sandholm T. Safety aspects of *lactobacillus* and *bifidobacterium* species originating from human oro-gastrointestinal tract or from probiotic products. Microb Ecol Health Dis. 2002;14(4):234–41.

[19] Østergaard L, Valeur N, Tuxen CD, Bundgaard H, Iversen K, Moser C, et al. Content area Ugeskr Læger. 2022;184:V10210751. 35319455

[20] Andersen MH, Holle SLK, Klein CF, Bruun NE, Arpi M, Bundgaard H, et al. Risk for infective endocarditis in bacteremia with Gram positive cocci. Infection. 2020;48(6):905–12. 32844380 10.1007/s15010-020-01504-6

[21] Hardie JM, Whiley RA. Classification and overview of the genera *Streptococcus* and *Enterococcus*. J Appl Microbiol. 1997;83(S1):1s–11s. 28621895 10.1046/j.1365-2672.83.s1.1.x

[22] Gaucher F, Bonnassie S, Rabah H, Marchand P, Blanc P, Jeantet R, et al. Review: adaptation of beneficial *Propionibacteria*, *Lactobacilli*, and *Bifidobacteria* improves tolerance toward technological and digestive stresses. Front Microbiol. 2019;10:841. 31068918 10.3389/fmicb.2019.00841 PMC6491719

[23] Escrihuela-Vidal F, López-Cortés LE, Escolà-Vergé L, De Alarcón González A, Cuervo G, Sánchez-Porto A, et al. Clinical features and outcomes of *Streptococcus anginosus* group infective endocarditis: a multicenter matched cohort study. Open Forum Infect Dis. 2021;8(6):ofab163. 34189163 10.1093/ofid/ofab163 PMC8231368

[24] Bolyen E, Rideout JR, Dillon MR, Bokulich NA, Abnet CC, Al-Ghalith GA, et al. Reproducible, interactive, scalable and extensible microbiome data science using QIIME 2. Nat Biotechnol. 2019;37(8):852–7. 31341288 10.1038/s41587-019-0209-9 PMC7015180

[25] Faust K, Raes J. CoNet app: inference of biological association networks using Cytoscape. F1000Res. 2016;5:1519. 27853510 10.12688/f1000research.9050.1 PMC5089131

[26] Romero Gomez M, Peinado Esteban A, Sobrino Daza J, Saez Nieto J, Alvarez D, Pena Garcia P. Prosthetic mitral valve endocarditis due to *Ochrobactrum anthropi*: case report. J Clin Microbiol. 2004;42(7):3371–3. 15243121 10.1128/JCM.42.7.3371-3373.2004 PMC446288

[27] Vaca DJ, Dobler G, Fischer SF, Keller C, Konrad M, von Loewenich FD, et al. Contemporary diagnostics for medically relevant fastidious microorganisms belonging to the genera Anaplasma,Bartonella,Coxiella,OrientiaandRickettsia. FEMS Microbiology Reviews. 2022. 10.1093/femsre/fuac013 PMC9300619 35175353

[28] Radaic A, Kapila YL. The oralome and its dysbiosis: new insights into oral microbiome-host interactions. Comput Struct Biotechnol J. 2021;19:1335–60. 33777334 10.1016/j.csbj.2021.02.010 PMC7960681

[29] Santibáñez P, García-García C, Portillo A, Santibáñez S, García-Álvarez L, de Toro M, Oteo JA. What does 16S rRNA gene-targeted next generation sequencing contribute to the study of infective endocarditis in heart-valve tissue? Pathogens. 2021;11(1):34-46. 10.3390/pathogens11010034. 10.3390/pathogens11010034 PMC8781873 35055982

[30] Callahan BJ, McMurdie PJ, Rosen MJ, Han AW, Johnson AJA, Holmes SP. DADA2: high-resolution sample inference from Illumina amplicon data. Nat Methods. 2016;13(7):581–3. 27214047 10.1038/nmeth.3869 PMC4927377

[31] Ziemski M, Wisanwanichthan T, Bokulich NA, Kaehler BD. Beating Naive Bayes at Taxonomic Classification of 16S rRNA Gene Sequences. Frontiers in Microbiology. 2021;Volume 12 - 2021. 10.3389/fmicb.2021.644487 PMC8249850 34220738

[32] Quast C, Pruesse E, Yilmaz P, Gerken J, Schweer T, Yarza P, et al. The SILVA ribosomal RNA gene database project: improved data processing and web-based tools. Nucleic Acids Res. 2013;41(Database issue):D590–6. 10.1093/nar/gks1219 PMC3531112 23193283

[33] Gwak H-J, Rho M. Data-driven modeling for species-level taxonomic assignment from 16S rRNA: application to human microbiomes. Front Microbiol. 2020;11:570825. 10.3389/fmicb.2020.570825. 10.3389/fmicb.2020.570825 PMC7688474 33262743

[34] Williams RJ, Howe A, Hofmockel KS. Demonstrating microbial co-occurrence pattern analyses within and between ecosystems. Front Microbiol. 2014;5:358. 10.3389/fmicb.2014.00358. 10.3389/fmicb.2014.00358 PMC4102878 25101065

[35] Huang Y, Wang Z, Ma H, Ji S, Chen Z, Cui Z, et al. Dysbiosis and Implication of the Gut Microbiota in Diabetic Retinopathy. Front Cell Infect Microbiol. 2021;11:646348. 10.3389/fcimb.2021.646348. 10.3389/fcimb.2021.646348 PMC8017229 33816351

[36] Bokulich NA, Kaehler BD, Rideout JR, Dillon M, Bolyen E, Knight R, et al. Optimizing taxonomic classification of marker-gene amplicon sequences with QIIME 2’s q2-feature-classifier plugin. Microbiome. 2018;6(1):90. 29773078 10.1186/s40168-018-0470-z PMC5956843

[37] Okamoto M, Naito M, Miyanohara M, Imai S, Nomura Y, Saito W, et al. Complete genome sequence of *Streptococcus troglodytae* TKU31 isolated from the oral cavity of a chimpanzee (*Pan troglodytes*). Microbiol Immunol. 2016;60(12):811–6. 27921343 10.1111/1348-0421.12453

[38] Mattos-Graner RO, Jin S, King WF, Chen T, Smith DJ, Duncan MJ. Cloning of the *Streptococcus mutans* gene encoding glucan binding protein B and analysis of genetic diversity and protein production in clinical isolates. Infect Immun. 2001;69(11):6931–41. 11598068 10.1128/IAI.69.11.6931-6941.2001 PMC100073

[39] Patel NK, Patel V, Vasava S. Evaluation of biofilm formation in bacterial isolates from cardiac valve infections. J Heart Valve Dis. 2025;30:245–8.

[40] Sullivan MJ, Leclercq SY, Ipe DS, Carey AJ, Smith JP, Voller N, et al. Effect of the *Streptococcus agalactiae* virulence regulator CovR on the pathogenesis of urinary tract infection. J Infect Dis. 2017;215(3):475–83. 28011914 10.1093/infdis/jiw589 PMC6455028

[41] Jang YR, Song JS, Jin CE, Ryu BH, Park SY, Lee SO, et al. Molecular detection of Coxiella burnetii in heart valve tissue from patients with culture-negative infective endocarditis. Med (Baltimore). 2018;97(34):e11881. 10.1097/MD.0000000000011881. 10.1097/MD.0000000000011881 PMC6112960 30142785

[42] Fotoglidis A, Pagourelias E, Kyriakou P, Vassilikos V. Endocarditis caused by unusual *Streptococcus* species (*Streptococcus pluranimalium*). Hippokratia. 2015;19(2):182. 27418771 PMC4938113

[43] Ismail A, Yogarajah A, Falconer JL, Dworakowski R, Watson S, Breeze J, et al. Insights into microorganisms, associated factors, and the oral microbiome in infective endocarditis patients. Front Oral Health. 2024;5:1270492. 38665315 10.3389/froh.2024.1270492 PMC11043546

[44] Armstrong GP. Infective Endocarditis MSD Manual Professional Edition: MSD Manual; 2024 [updated 2024/07. Available from: https://www.msdmanuals.com/professional/cardiovascular-disorders/endocarditis/infective-endocarditis.

[45] Krcmery V, Demitrovicova A, Hricak V, Kisac P. Endocarditis due to gram-negative bacteria. Int J Infect Dis. 2010;14:e359. 20153234 10.1016/j.ijid.2009.08.022

[46] Kappel BA, Lehrke M. Mikrobiom, Diabetes und Herz: neue Zusammenhänge? Herz. 2019;44(3):223–30. 30847506 10.1007/s00059-019-4791-x

[47] Rabinovich D, Yazigi M, Yazigi A, Zainah H. Infective endocarditis due to *Haemophilus parainfluenzae*: a case report and review of the literature. Journal of Brown Hospital Medicine. 2023;2(3):77739. 40026470 10.56305/001c.77739 PMC11864410

[48] Saha S, Joskowiak D, Marin-Cuartas M, De La Cuesta M, Weber C, Luehr M, et al. Cutibacterium acnes infective endocarditis-an emerging pathogen. Eur J Cardiothorac Surg. 2024;66(6):ezae422. 10.1093/ejcts/ezae422. 10.1093/ejcts/ezae422 39585651

[49] Barril BP. Francisella, Brucella and Pasteurella. In: Rezaei N, editor. Encyclopedia of infection and immunity. Oxford: Elsevier; 2022. p. 673–84.

[50] Chambers ST, Murdoch D, Morris A, Holland D, Pappas P, Almela M, et al. HACEK infective endocarditis: characteristics and outcomes from a large, multi-national cohort. PLoS ONE. 2013;8(5):e63181. 23690995 10.1371/journal.pone.0063181 PMC3656887

[51] Baddour LM, Wilson WR, Bayer AS, Fowler VG Jr, Tleyjeh IM, Rybak MJ, et al. Infective endocarditis in adults: diagnosis, antimicrobial therapy, and management of complications: a scientific statement for healthcare professionals from the American Heart Association. Circulation. 2015;132(15):1435–86. 26373316 10.1161/CIR.0000000000000296

[52] Fournier P-E, Thuny F, Richet H, Lepidi H, Casalta J-P, Arzouni J-P, et al. Comprehensive diagnostic strategy for blood culture-negative endocarditis: a prospective study of 819 new cases. Clin Infect Dis. 2010;51(2):131–40. 20540619 10.1086/653675

[53] Caliman-Sturdza OA. Enteroccocus and Endocarditis. In: Téllez-Isaías G, Graham D, El-Ashram S, Gray L, editors. Enterococcus - Unveiling the Emergence of a Potent Pathogen. London: IntechOpen; 2024.

[54] Nørskov-Lauritsen N, Riber LPS, Dzajic E, Prangsgaard K. Three cases of *Cutibacterium avidum* prosthetic valve infective endocarditis at a single heart center. Int J Infect Dis. 2024;146:107099. 38762047 10.1016/j.ijid.2024.107099

[55] McGhie D, Hutchison JG, Nye F, Ball AP. Infective endocarditis caused by *Streptococcus mutans*. Br Heart J. 1977;39(4):456–8. 869980 10.1136/hrt.39.4.456 PMC483257

[56] Riaz Rajoka MS, Shi J, Mehwish HM, Zhu J, Li Q, Shao D, et al. Interaction between diet composition and gut microbiota and its impact on gastrointestinal tract health. Food Sci Hum Wellness. 2017;6(3):121–30.

[57] Altveş S, Yildiz HK, Vural HC. Interaction of the microbiota with the human body in health and diseases. Biosci Microbiota Food Health. 2020;39(2):23–32. 32328397 10.12938/bmfh.19-023 PMC7162693

