# Supplementary material for: Metagenomic insights into microbial community alterations and co-occurrence networks in infective endocarditis
Source: Genomics Inform. 2025 Dec 2;23:25. doi: 10.1186/s44342-025-00059-y (PMC12670860; doi:10.1186/s44342-025-00059-y)
Supplement: Supplementary file 1 — Supplementary Material 1: Table S1. Table summary before filtering. Table S2. Details of frequency based on frequency in per sample. Table S3. Feature counts in each sample. Table S4. Feature details in each sample. Table S5. Demultiplexed sequence counts summary. Table S6. Sequence counts in per sample. Table S7. Demultiplexed sequence length summary. Table S8. Table summary after filtering. Table S9. Frequency per sample after filtering. Table S10. Kruskal–Wallis results for alpha diversity. Table S10. The correlations between identified families based on Spearman's correlation. Table S11. The correlations between identified genera based on Spearman's correlation. Figure S1. Demultiplexed sequence counts summary plot (A) Forward Reads Frequency, (B) Reverse Reads Frequency. Figure S2. Quality plot for forward reads (These plots were generated using a random sampling of 10000 out of 11599552 sequences without replacement. The minimum sequence length identified during subsampling was 300 bases. Outlier quality scores are not shown in box plots for clarity.) Figure S3. Quality plot for reverse reads (The plot at position 131 was generated using a random sampling of 10000 out of 11599552 sequences without replacement. The minimum sequence length identified during subsampling was 300 bases. Outlier quality scores are not shown in box plots for clarity) [file 44342_2025_59_MOESM1_ESM.docx]

Metagenomic Analysis and Investigation of Microbiome Dysbiosis and Co-occurrence Network in Infective Endocarditis Patients

Zahra Abedi^1*^, Amirreza Abbasi^2^, Negar Sadat Soleimani Zakeri^3^, Helia Jangi^4^

^1^ Department of Biotechnology, College of Science, University of Tehran, Tehran, Iran.

^2^ Department of Health science, University of Eastern Piedmont, Novara, Italy

^3^ Department of Software Engineering, Engineering and Architecture Faculty, Istanbul Nişantaşi University, Istanbul, Turkey.

^4^ Department of Science and Technological Innovation, University of Eastern Piedmont, Novara, Italy.

**ORCID IDs:**

Zahra Abedi: 0000-0002-6750-0208

Amirreza Abbasi: 0000-0001-5437-679X

Negar Sadat Soleimani Zakeri: 0000-0002-3343-9904

Helia Jangi: 0000-0007-8782-6989

**Corresponding Author:**

***Correspondence Author**

| Zahra Abedi.  Department of Biotechnology, College of Science, University of Tehran, Tehran, Iran.  E-mail: [abedizahra59@yahoo.com](mailto:abedizahra59@yahoo.com)  Tel: +989120422155 |
| --- |

# Supplementary Figures and Tables

## Supplementary Tables

| **Metric** | **Sample** |
| --- | --- |
| Number of samples | 27 |
| Number of features | 774 |
| Total frequency | 23998 |

**Table S1.** Table summary before filtering.

| **Metric** | **Frequency** |
| --- | --- |
| Minimum frequency | 0 |
| 1st quartile | 123.5 |
| Median frequency | 530 |
| 3rd quartile | 1109.5 |
| Maximum frequency | 5698 |
| Mean frequency | 888.8 |

**Table S2.** Details of frequency based on frequency in per sample.

| **Sample ID** | **Feature Count** |  | **Sample ID** | **Feature Count** |
| --- | --- | --- | --- | --- |
| SRR13685051 | 5698 |  | SRR13685052 | 403 |
| SRR13685043 | 2443 |  | SRR13685053 | 328 |
| SRR13685066 | 2035 |  | SRR13685063 | 266 |
| SRR13685050 | 1861 |  | SRR13685041 | 251 |
| SRR13685060 | 1455 |  | SRR13685046 | 196 |
| SRR13685061 | 1451 |  | SRR13685045 | 192 |
| SRR13685055 | 1138 |  | SRR13685054 | 55 |
| SRR13685040 | 1081 |  | SRR13685057 | 51 |
| SRR13685049 | 1021 |  | SRR13685062 | 27 |
| SRR13685059 | 1009 |  | SRR13685047 | 3 |
| SRR13685042 | 951 |  | SRR13685065 | 2 |
| SRR13685048 | 930 |  | SRR13685044 | 0 |
| SRR13685056 | 621 |  | SRR13685064 | 0 |
| SRR13685058 | 530 |  | ------ | ------ |

**Table S3.** Feature counts in each sample.

| **Feature** | **Frequency** | **Number of features in per sample** |
| --- | --- | --- |
| 08eea61bcfcdfd598d8c98ff47991041 | 808 | 12 |
| 53ae69208f647b9c7668cb147f6b8bc6 | 761 | 12 |
| 9d67a639afa54522a90f3a0a832d3a43 | 679 | 5 |
| 196eb533560c33ae39fcbc2daa43b3b7 | 579 | 12 |
| 509ae0847ed40b2a88306c41c50b516a | 577 | 4 |
| b86caf590725850ceecdd9306d3f9e78 | 525 | 9 |
| 01dd2f570f421add71e52441fe83d18b | 479 | 10 |
| af1afb5bc9892086d6d754ade2c0ed28 | 475 | 4 |
| c77dbfb122e64372fbc577bde14dea6c | 462 | 9 |
| 7b743444fde0237b109ce6aff717614b | 456 | 10 |
| 75067ca82612c198a2faeda20fae6657 | 454 | 4 |
| f706c5a962c4130627b34e67432f1ee2 | 452 | 3 |
| 7cbb41db42592fed06d0b3720423326c | 416 | 4 |
| 0acbb864b82de516022713235f8b8b69 | 402 | 11 |
| b0f10dd7265cad689ff6b5ffda299612 | 400 | 8 |
| b16a63e396a0479cc5cbfd1f87959bc6 | 400 | 7 |
| 67712c76c75d8c1c763145e8ffdfc638 | 387 | 8 |
| f8dc2a7e9edc1771c6d9a4659a5e8a63 | 364 | 9 |
| 2e225474dd8a1892c254eba4627a2e2c | 340 | 9 |
| 912a40584ad27cea979d790238ef2130 | 340 | 5 |
| db8e43e5828b9abf1f7742eec83b0e24 | 336 | 10 |
| 7982d84c708655eeb1bba23c586f6c48 | 334 | 1 |
| 3eb0ca7d6f33defef1777ebd3ba7f71c | 296 | 6 |
| 1f924e68837996f15a819585dd87a9e6 | 267 | 2 |
| e5618f343cb65eba20fe2c0c55e4b0dd | 253 | 1 |
| 6ff20902e06c8e31a56153de76fc3e5c | 231 | 5 |
| 403865247465d7fd6739203cc57219c8 | 227 | 9 |
| ecb5b374225d64c58133756cc3900207 | 224 | 1 |
| de444577b482ba4cc1dce20904975fcc | 190 | 1 |
| 8820d4d442cafb70236e49eeb3acd41e | 188 | 7 |
| b0947d27e1de8461de1f3c9ef27c37d3 | 181 | 5 |
| 2595daeadace9103b60785a1feb13a15 | 171 | 1 |
| 4e7cb2fa21ec19eb5d9857883fb83ed1 | 170 | 3 |
| 7d7011b8499101c94f65196a9952b21d | 165 | 1 |
| edf19c61553cf2638547dd784f612546 | 159 | 3 |
| 9de0adba95b314202ea0b9e673fa9da3 | 152 | 2 |
| 5062339089ac4eb7011a3727520d09e5 | 147 | 5 |
| fe3a25710a783ce431b28657def41931 | 141 | 1 |
| 39c249f8754bab7b99c3ff669853894d | 138 | 2 |
| e63928c521500d853f6636d2f9759aa7 | 138 | 3 |
| ee0f89056acf81ec36203794aac5aed4 | 130 | 1 |
| 6af36dba17e937cfc50b58da5434d654 | 129 | 1 |
| 17576d030b1c4131be94ff1bf8b06746 | 127 | 1 |
| f01455ac4b13b449bf175010a5a1cc4c | 121 | 1 |
| a33680efe1ba3111d9f4bdc08071fac3 | 120 | 1 |
| fedbe2752514d6d7a71d98e43cd6b6ff | 119 | 1 |
| 4beeccb0bea60f7fa26c11589744a02e | 118 | 4 |
| 0d139b4dca3cd9043ff35046a5ca740a | 117 | 1 |
| cfada3737a4e454b18de0986b6e138c5 | 112 | 1 |
| 8b26452f0b30dd2f0ac5e4af9b40fb4f | 107 | 3 |
| 6dc137b3e647e57ad0cf48da9a2e82d5 | 104 | 3 |
| 47bb4c430bf663d9ab73028d4b8f20b7 | 103 | 1 |
| f8661caa3532ceb1a13b11ce070adb0d | 98 | 2 |
| dc835b54d9e2d29a0a62458230933362 | 97 | 1 |
| 1573839f5267e2d4421b5c16004dce8e | 94 | 1 |
| a4d977142cdde604f89d7600825c8591 | 93 | 1 |
| b67b14c07e79ee9644a76f000c83e99e | 88 | 1 |
| e7d9cbc6eb8b9a32632184e50d855ee6 | 88 | 1 |
| 41253734ef0e929ed289ac25e939c3ef | 86 | 1 |
| 75ca605e51edea2ad5b2774f9522005b | 86 | 2 |
| 8577362d60000b351cda39b1eedc54bb | 83 | 1 |
| cff28d85ac15aa73c8b5fbbe329e9627 | 79 | 3 |
| 8310e56b659062e56788598c0d41cf96 | 75 | 1 |
| f31bac5390d272fbb21d7fe880e9e0a8 | 75 | 1 |
| e387d6e3895fb7902ea7b85d3cf4fa19 | 74 | 1 |
| fe48fc2f124139bed8efce0165b3fafb | 74 | 1 |
| 268e31b50663276cd8ae22a418f730d1 | 73 | 1 |
| 329905a409c06c16ef45c400810222fe | 72 | 1 |
| 2b8a6831fc4b103d392e84db0f7b9687 | 71 | 1 |
| 2d9044e3b31e85c5f0191ccad30cf5ff | 71 | 1 |
| ac05e78ba1154cc007a4c43e72949ca4 | 71 | 1 |
| e54100274301627fa65210c6eb98349f | 70 | 1 |
| 670ef960b61faa716dacb828b5a6b316 | 69 | 1 |
| 22b23e86109bcc41bc324bcc5306b745 | 68 | 1 |
| 846cba4843cfe1ec35b17afdff1b8f19 | 68 | 1 |
| dcf592b004c824c665310af529ea069c | 67 | 2 |
| ac73d2f3a35fd48b1dd8750e4325d5f7 | 66 | 2 |
| 26a62b00a8f94329589a3fd2b1fbf491 | 65 | 1 |
| b17efb0267aa659ac75a8d51fa38da54 | 63 | 1 |
| 64875c1568224f364e70f9fca0e059c3 | 62 | 1 |
| 78111515d048f4ba02ec5cdc55674ada | 62 | 1 |
| caa7b43cb5295954e86aad8042bdabcb | 62 | 1 |
| 27352db429b2394499e58e511881145c | 61 | 3 |
| e9ec89346cfe075f63f339cc18b8ec56 | 61 | 1 |
| 4c009e622420fb92b6591d073529015e | 60 | 1 |
| 8a80e287871f90c176e1592f53d9d378 | 60 | 1 |
| db81dfe4bd3056abb1ca4367bda32f20 | 60 | 2 |
| ab4fb8f643df23c8ffc484511283833f | 58 | 1 |
| 85b720103f99ef3c468e68600d2f6103 | 57 | 1 |
| 00f36de37ee9ac726fae7cd2f7c36055 | 56 | 1 |
| 0cf8492ae9a731bba948fd54267d4739 | 56 | 1 |
| 128e493735fddff715790aeab0868986 | 56 | 1 |
| a55fe439139f44fa8b02bca43d603d95 | 56 | 1 |
| c97bfcf33e2b3eb9e78f787fc5317927 | 56 | 2 |
| 2dec17cf22b252c1b328a86f79702c34 | 50 | 1 |
| 50eecbd8435c15d57e9fb6628caf7dae | 48 | 3 |
| d4f627c8dd3f9e7d111574ab22fd1926 | 48 | 1 |
| 934198cd990b82871d9384414ccff002 | 47 | 2 |
| b6f745814e8d1aa8fcaea821785da5b0 | 47 | 1 |
| f4f7cb9df5b59bb095715dc03092406b | 46 | 1 |
| 1a89ce94c7df4e0e9ea6a321715a3d4b | 45 | 1 |
| 405f3f7e482144398ac5f3f19eb877da | 45 | 1 |
| d7372a569ed6b6a80b24dc44c321f066 | 45 | 1 |
| 54b008e95f2764f8c190c7fd4ccfe76a | 43 | 1 |
| 49b1ff8644a18fbeda971fddbb9956ce | 41 | 1 |
| c6fec13db0050049d53dc427f18f0568 | 40 | 1 |
| d17839c7346856906149a688652a51ad | 40 | 1 |
| 49871fca208e08a01be024aae7f543d3 | 39 | 1 |
| 5f8d79f12407dc20cf73434272ede963 | 39 | 1 |
| 72f27a91c96dfd92e1ae5f2b610f05e5 | 39 | 1 |
| 1d2903428f5344b145e98565b290c0da | 36 | 1 |
| 3dd02e2bb718502d1bc941538450c2f9 | 36 | 1 |
| 836e2ac044a700292239ff4279f7406a | 36 | 1 |
| bba24ea3747579080f19a49dffaa69f4 | 36 | 2 |
| d3d1dcbeffaffdae7bb2fa4174597a91 | 36 | 1 |
| defd38d2320ca1bafc2f418c9d0bdb99 | 36 | 1 |
| 27a9f555e8e7dc81f31f712ec926e89f | 35 | 1 |
| ae991fd0b40f28998d9a1b9978aa2743 | 34 | 1 |
| aec20e19903e4cebb22e408e010cc574 | 34 | 1 |
| aefbb0755f7ccf9d061756acdc8052bb | 34 | 1 |
| 5e2a176fe4377ee3ce5cd7dc01d3a9bb | 33 | 1 |
| cdc2c5d1a8da0e214e65166d8a50919f | 33 | 1 |
| 0db4f556b6c6fc6f17135333a3c1ebfb | 31 | 1 |
| 12857d434280cedd178d2e77c7b3a06a | 31 | 1 |
| a00e8366c7830638948338d10bdacad4 | 31 | 1 |
| a9b0495727e8e70e693a8c1833d45e0d | 31 | 1 |
| 7002b1d5d31ec6f1ee9524ae0cb26889 | 30 | 1 |
| 93b5195d85d777c55c356f9ed9b72ba7 | 30 | 1 |
| e3e60ab9b5cad7631b0ab7c3d2b25934 | 30 | 1 |
| 122c1a6c65deac42c32f119e8f7e2710 | 29 | 2 |
| 339958bdebbfb90fc212a535060320d8 | 29 | 1 |
| 488c56323222c900dba73e8c2dca541d | 29 | 2 |
| 5393e1466802eff82d2a272ccf323583 | 29 | 2 |
| 5a96e5f08984589c53866fa2013e5e27 | 29 | 1 |
| 5e8eed7204883f3ae1f84599438b8641 | 29 | 1 |
| a9eba852d0f0e901932e1c658ff53a76 | 29 | 1 |
| f189112cca5391166b5d42c0453360ce | 29 | 1 |
| 19abb6279dacdb17e8cc3c5fe2d38f3c | 27 | 1 |
| 8909c06ff9fcb2f08d4d4b46fe6c1125 | 27 | 1 |
| 9767cf3233e48a766f715ccc5fc66bca | 27 | 2 |
| 36d88efc65acb89e3c0a79ca9750d821 | 26 | 1 |
| 377e9258cdc2160736ec27a72e8773b3 | 26 | 2 |
| 644035d5a1fbe3578896be3911837ab3 | 26 | 2 |
| cb8fda21d1902962d911601841d4337b | 26 | 1 |
| 1123322ca79ef2380cf64282c33b5466 | 25 | 2 |
| 23687e35ec82fcab89402bafa4284105 | 25 | 1 |
| 48748f4a5462422e1693e2b462944954 | 25 | 1 |
| d478d3916bfaee460c57e532ceaf46ff | 25 | 1 |
| d72d0720eb0920f2c511d713df479d26 | 25 | 2 |
| edbc32582fb5bc5eef10bf8f284861d8 | 25 | 1 |
| eeff507c2804a40cb356b5bd807b3b24 | 25 | 1 |
| 0977c2d16ed4ee8d5cb64c7364e82ada | 24 | 1 |
| 0cce309239943d6b3f9fa28d220855c3 | 24 | 1 |
| 8ffed2f9ee7f35667bf2065dbd0a1d56 | 24 | 1 |
| de6c029f0d649d91b878847eff917fef | 23 | 1 |
| a5d664f08ba8cc58496784de2143b647 | 22 | 1 |
| eae636df3e944bce0d0aceaff65b3c84 | 22 | 1 |
| 1294f9b149adf6996760618c567145c1 | 21 | 1 |
| 26930fa4d28e8fc50f4ee33be27d9085 | 21 | 1 |
| 5bd9555cc43cf001c32bc0f038392622 | 21 | 1 |
| 06570bb7323803b8856603218e27c3f1 | 20 | 1 |
| 3cf707d0c65d26a76cd5e41df6ccaa11 | 20 | 1 |
| bdd04e4984534cd15b637df25f3a9286 | 20 | 1 |
| be5fe70bcc4eb1cf53114b4ab196bc1b | 20 | 2 |
| d9216704e71ed482b328e9fec749c3ea | 20 | 1 |
| 194e47cdf2957ad15d0053b20e87e631 | 19 | 1 |
| 2c583db910ecf9b76a33d8d3d5631ba8 | 19 | 1 |
| 3b509938a12389cc75c0ff0fe64861e5 | 19 | 1 |
| 562053da9e9047b47aff361e9b1df193 | 19 | 1 |
| ae264002af95f943c4edd2e318d8a7e5 | 19 | 1 |
| c7b95a68b6cc6682a98b8e51869c91e5 | 19 | 1 |
| 61813005995caf1f27e317f5849a22de | 18 | 1 |
| 91bf30e68f37f60f1c9c9cfa6cb7f706 | 18 | 1 |
| bb5bb211f2a5a6fbfd3e39b393f44f52 | 18 | 1 |
| ecddf1fed975addff335fe05185db2cd | 18 | 1 |
| 50935064959af50260565e41fc9c615c | 17 | 1 |
| 5ece5c19ed65bf06e1ad893886f6f137 | 17 | 1 |
| 6be01dd83e4d93d80a3fe1daf1ea381c | 17 | 1 |
| 7e0ee65f91c569b4adaa0379cb4d27dd | 17 | 1 |
| 8620a5cacc1973ef0679af05c5375555 | 17 | 1 |
| 8b6a7cebaba88528302acc7a63c12d3c | 17 | 2 |
| 91ec4bc41f626986b693b616b7b0f966 | 17 | 1 |
| 99e272f34b699d57be7f8360742214d2 | 17 | 1 |
| 9b22cefb4358ea6eb21d1ab5d8057625 | 17 | 1 |
| d80672acb79ed19cbbc5e476a001e145 | 17 | 1 |
| e5f5fe96705d0581ab796b9f1ed7f7c5 | 17 | 1 |
| febc3cc7a05d4afbc8bd0ba0e87b8a3f | 17 | 1 |
| 0430a5e4627d2089758d9b9ebdf4bb3b | 16 | 1 |
| 1bad5a12117794f0019627068a1aace0 | 16 | 1 |
| 307c5172e80e8898e3e6034905735753 | 16 | 1 |
| 647a4ce4fc473986a562afd42a084ad0 | 16 | 1 |
| 74e7e99fe58a47af418550961aa44ab7 | 16 | 1 |
| 83f1eadc73e0637a9207d24a9c91b4c6 | 16 | 1 |
| 15c31b2a384c1029cccbbd46c19703c1 | 15 | 1 |
| 1eaf7960027be8b7594724315dcf609f | 15 | 1 |
| 4b64937c25a66d72967f816f71e9db4c | 15 | 1 |
| 6116b3a69468da35b585fdd21fc5452f | 15 | 1 |
| e535eecc01f7ac8f463212a8d8e76ae3 | 15 | 1 |
| 00923bd66192a407b99b07470908a061 | 14 | 1 |
| 0497a70cb97f03865b25a3660791ce99 | 14 | 1 |
| 138d69ce6211ff397ab272facb29f4d0 | 14 | 1 |
| 16a157c8fd015a8aa1346f23c53ecf9e | 14 | 1 |
| 2088176bf47ccb5b021a37609f7aeb03 | 14 | 1 |
| 2208a20e91d7a0051acded2e0c6301c4 | 14 | 1 |
| 3246330e8f5174bd01fb7a3348f3bd75 | 14 | 1 |
| 3e917d4caa98698b411e44a4be084ab4 | 14 | 1 |
| 607dd40d159a50abc48a6afc307f8554 | 14 | 1 |
| 6413c2feddd3a558831a538d8f1bf0b9 | 14 | 1 |
| 7455a07f588433816e31f8b6b54491b1 | 14 | 1 |
| 8bae3b92cd24b26e51ea4e3bfe51ee5f | 14 | 1 |
| 9ea84c82a10441570983ed48931da5c7 | 14 | 1 |
| b448c35560084e90e036b3353303a383 | 14 | 1 |
| b784248c8feaed1ac76494a40c375cbe | 14 | 2 |
| cf01c588d57a9bfef88b0c79e078b814 | 14 | 1 |
| d720393d3f1785da6b8afbc1093df0ee | 14 | 1 |
| f3fbf1ec6af0e2f293eb0cab8075efe5 | 14 | 1 |
| 1fc8ae48fa48900ff9ccaebe84ab3da9 | 13 | 2 |
| 309180ac774e13ea0ec0669e7928ae01 | 13 | 1 |
| 3095efa0a2cdf6587ca4c35917bc6415 | 13 | 2 |
| 3161be6f06ef4bb64cc59f06f2fdfc44 | 13 | 1 |
| 7ff696b1ead217b7ae4b52c18817616f | 13 | 1 |
| 8a0cb7f344cc1d5b06630cb127c4d2ba | 13 | 1 |
| 94d78fd4527655179193601e960b48f9 | 13 | 1 |
| 9ac8b1adedd6d57ed9d62ffae3cc6bb6 | 13 | 1 |
| 9af3282d5cb44c01b059606f36debe44 | 13 | 1 |
| aa7877227b355d7e7c9e3fccfae7f1be | 13 | 1 |
| b456f6fbc35f0da87bcb512ebcc22cee | 13 | 1 |
| c8fe52806f3b3667d48dc5b0f20f14e2 | 13 | 1 |
| cd066a382e82d00d558122e01e3262b1 | 13 | 1 |
| ce895d63e048a8c64a951cb1527c8b94 | 13 | 1 |
| ee597c5200319968379410c5226822e9 | 13 | 1 |
| f2a096dd529a2e85433a38b0fd066a4a | 13 | 1 |
| 19b558367fda5f71ed67e4f72954bafc | 12 | 1 |
| 846ccc0c260cbf8d0e47b3b8246dc0fa | 12 | 1 |
| 8ac45be3c04e454f182d1d3d15d10c7a | 12 | 1 |
| a4cfb1fa62f0016baff8953210855901 | 12 | 1 |
| c130acf908ece6ee901251c4bef38ae1 | 12 | 1 |
| d0edebc409514dc7078e012a733cf328 | 12 | 2 |
| e304ffdf4634594d7fce54f7c84de365 | 12 | 1 |
| f4107fc8b37ccdad4a0ea439f1a05c73 | 12 | 1 |
| 09ccbae93f4606be279e4710b6593486 | 11 | 1 |
| 199360a2cca456ca4403bc5a7ae98efc | 11 | 1 |
| 1ee49e352cb8f549cb3e8bf05bf2d10e | 11 | 1 |
| 3c7cc7b76a8ee28f28a04f5ff6c2fea8 | 11 | 1 |
| 3f6ea1d924b50fc1ac22b092d57d423b | 11 | 1 |
| 46c5dac62496680ea8ce886c67da2533 | 11 | 1 |
| 4842b79850e8072dde3dcab417517ad2 | 11 | 1 |
| 6979ad423795e84d4d16a39becf3a745 | 11 | 1 |
| 6a621c875c9136f6a5cff1ec36276ead | 11 | 1 |
| 7173216328a5a930c03d4b98642c1e31 | 11 | 1 |
| 836c68262692956959e95e48e8bdb757 | 11 | 1 |
| 964d59594cf63319429926f4a4070de2 | 11 | 1 |
| 9e79d3b879920e45ec386b9668518171 | 11 | 1 |
| c39e6c2a3fbc4fea3b9b3858abca628e | 11 | 1 |
| fb08850474c4fb9644bb36624ab245b9 | 11 | 1 |
| 032943c455b5315e59ace01d8510caa2 | 10 | 1 |
| 0b4093d9805c0dc6773c611c188bfee0 | 10 | 1 |
| 0ca0253f97169423863c08dd2f27dd9b | 10 | 1 |
| 1ce3e44c0189614d28b6a50c6a400fdb | 10 | 1 |
| 345d7694d4e29b2720242543ff88a2f1 | 10 | 1 |
| 34b712df74ca4333025c942036769638 | 10 | 1 |
| 3b7fba5a0de555db4ce277f5836abc2b | 10 | 1 |
| 44377b7c2a7653980fab56995347b7d2 | 10 | 1 |
| 58a2d97d7bec5de11ae4fff53053ceca | 10 | 1 |
| 5c7c0da73fdb393466a787dc3a925ff0 | 10 | 1 |
| 5e8d4fdd17d8343a57ec0cf9838d9954 | 10 | 1 |
| 73aa58c98b59bc668391dc70eea06467 | 10 | 1 |
| 746c71cc381ed3774747145ecb997231 | 10 | 1 |
| 78d6d93135ce459744b7ba35a5a10449 | 10 | 1 |
| 891d74caacb7391aa042d2902a296b7e | 10 | 1 |
| 906810d624eef54b59d2f530497baaec | 10 | 1 |
| 9d4248585848f798d0e6770311efffd3 | 10 | 1 |
| b7c82a0cc8b970dc3ef1f9b594f835b2 | 10 | 1 |
| ccb9b330b0211178d68a72bc2d0ddeda | 10 | 1 |
| d442434af4b6605593726bb097278141 | 10 | 1 |
| df9dc004089c20fe7355c24b51b581e7 | 10 | 1 |
| f471d502958fe09f130942bd88fe8d52 | 10 | 1 |
| fc854fd1b29571da72c6b9c33352ceee | 10 | 1 |
| 04f642e3a0da5255dfc4b40ecad3903a | 9 | 1 |
| 06f9d4e4c4317c411be7dfa190555f6d | 9 | 1 |
| 093d1e93f83502a3dcb2b185050502b9 | 9 | 1 |
| 19f27f64bb5515dc1a57b2725b47bbac | 9 | 1 |
| 22f92bbc405bf81c615df647cb9fcb4d | 9 | 1 |
| 29b8670b208504e6e8303ebe9884e083 | 9 | 1 |
| 2b3f8c6012f641d259240771ca30c9fc | 9 | 1 |
| 42703082ea61b211b0d467c51396290a | 9 | 1 |
| 46e2487e3f86ce13778ee250b767840b | 9 | 1 |
| 52fb88b0c4f1ec33d10ed7f8a73b8fa8 | 9 | 1 |
| 89788fa8af0ab9755f83ad209688cac0 | 9 | 1 |
| 92ebb56e2c88f67021a0b112e3dae00a | 9 | 1 |
| 962420151312a29864ab0754c79fe1d1 | 9 | 1 |
| 9c1ababd416597aaababe06383b22c88 | 9 | 1 |
| a3b1300f97722932b32f68f65f66e75d | 9 | 1 |
| ccb7749e7a2290491d4a0c44676bba7a | 9 | 1 |
| d477cc842dae6031ad23cb66d05c3a50 | 9 | 1 |
| de7e0fb57b1dfc0c15db14168034a87b | 9 | 1 |
| ea0f854a252ab6f7af169482ec3c8884 | 9 | 1 |
| f510df71a6f579bd5b0c76cff7fbdca6 | 9 | 1 |
| f603729c6d0b9601218f9a27cc68b604 | 9 | 1 |
| f6971e4bddb3373122ecc6447a873bff | 9 | 1 |
| 0f516e241fbe4834085dd0080624efb5 | 8 | 1 |
| 23c31292aead469f8aacce6e1b356697 | 8 | 1 |
| 28025e4517a3344a3535d2cb7ba03a73 | 8 | 1 |
| 29db669bbe533317b35285d571540eae | 8 | 1 |
| 322217afabef9d542dc08b692eaa665b | 8 | 1 |
| 339c6561f1e2cc600886200e360c21d9 | 8 | 1 |
| 44980eebe2f4fdb7713818d41eba3981 | 8 | 1 |
| 453cfde66f12608486675b5aab83d97b | 8 | 1 |
| 4cc258866c2ae8c0cce3f80219752389 | 8 | 1 |
| 532ef40a878585d6c66fd701aac825f9 | 8 | 1 |
| 54f72ebf764e10f376eb9ff4ad57edf5 | 8 | 1 |
| 622ddef9e2666dcd9accf5c9cbf3a43d | 8 | 1 |
| 67868241d9cb1936907fd4d0495a0a14 | 8 | 1 |
| 682e34daf207e5f1c6ded528fa852d3e | 8 | 1 |
| 9865816ebf49315b897dfd4a9639c21f | 8 | 1 |
| b3016b30b20eb7e98eabbdacadaf96bc | 8 | 1 |
| c89e6a0adb75264f7e7626f61b968038 | 8 | 1 |
| cee51e856fabe719229b5926d46f1f1a | 8 | 1 |
| d78702f16c95ce8662ff9a2d37ed0513 | 8 | 1 |
| eb51ea42c39aa1d336348c1e7d7d8616 | 8 | 1 |
| efef63e3f892c82468c54f91a8bc605f | 8 | 1 |
| fc612bc9025f998b404c5e491a9b18fb | 8 | 1 |
| fd9e026259cd9e3280264fcdb7d84713 | 8 | 1 |
| 0fa588258f1edea8fdc3ff20ad358c5a | 7 | 1 |
| 10e3b94397c28cfe43a43988bcbb6d12 | 7 | 1 |
| 139e6e97273fc365e59c8d8ebffffc37 | 7 | 1 |
| 14b2eb36efc2ca1c7601d4f65dd9a1e9 | 7 | 1 |
| 15865ae319471e2fb77f86510c498bb4 | 7 | 1 |
| 19c096ba0c411a21606a67c8b347cf89 | 7 | 1 |
| 1e71962282aaaa9daf3b9e119affec82 | 7 | 1 |
| 22743386f452f8c25ff9a4d0ba8f7539 | 7 | 1 |
| 245a080888174b2d2be74d65477a2b3d | 7 | 1 |
| 2527ead78a7f586cf7f522ab5ecce12d | 7 | 1 |
| 2da17277564f4216499f748e6e61320c | 7 | 1 |
| 308e56275df8dbeffd66344b8128c066 | 7 | 1 |
| 3ffbda74d92c818fd18673ee97d0f2fa | 7 | 1 |
| 4531459341ddc18b97548d207a9ef350 | 7 | 1 |
| 46ab365aff6e8327230e2628896e9190 | 7 | 1 |
| 565e58cb7e087952b718564070477c9b | 7 | 1 |
| 5b10dd94b896f911e47018325deff8c3 | 7 | 1 |
| 6da0776414de7247b1f9864922c1fc14 | 7 | 1 |
| 71c7fdaffe835aa6489b4750b65f5bc9 | 7 | 1 |
| 763b96be33b83e734342c33ef6766e8d | 7 | 1 |
| 7986573152631417023966468a50764d | 7 | 1 |
| 7f7a3da964343eb3834244aef06c1e40 | 7 | 1 |
| 7fa8bdb47a57f734825a863109801658 | 7 | 1 |
| 8298e9e684b243cd66e3c93263086a70 | 7 | 1 |
| 84eee8a8f77a5c37f327087a081e1cb3 | 7 | 1 |
| 8c292ed5bd9ac19c075e310bf7f8ba01 | 7 | 1 |
| 94ec4f5ceb5081a1d3fd53605312c50b | 7 | 1 |
| 952770d9b26bd500d7207b3ca9999d70 | 7 | 1 |
| 9c4c156aef309467d41543b988d91f90 | 7 | 1 |
| 9cc4fc71e1ad70d1c0d2cd5bebe8920f | 7 | 1 |
| a1b521b6e1d5c0ea81c03e64ae1136fa | 7 | 1 |
| a38653083e02e2314aea17ac0e163f84 | 7 | 1 |
| ae7c292b0174bd0388b4f43250521e12 | 7 | 1 |
| b3c4a6a479a908799064ef8db75ece77 | 7 | 1 |
| ceac2ea748ebee4221e04a6a62f1059e | 7 | 1 |
| cfaa79245f37aae60d7122df02768730 | 7 | 1 |
| d0f81a118b5afb25eec8213da7c95d9b | 7 | 1 |
| d13e9e9992a6a5ee9b343cd07b1a7863 | 7 | 1 |
| e63d40a84db524072e456b48d02fa0a2 | 7 | 1 |
| e74becc858e78298df64587129675ef7 | 7 | 1 |
| f1d0e788185baf2d1e1e59e059cf44e2 | 7 | 1 |
| f2b16c44c5c61e17ffb19c585b4cebe3 | 7 | 1 |
| 0df7e517fb54a09b4049e7588c36b7c0 | 6 | 1 |
| 1a223286c590c609a80a2f992bab9057 | 6 | 1 |
| 1bec399647f29bf8d017618f69513c3e | 6 | 1 |
| 22375ed4cae7e7eb6ea64820acc7b34a | 6 | 1 |
| 249d93482a87fbc6d2498985a8d300d7 | 6 | 1 |
| 283e35ea444c9e2764b7cb842c1b7f44 | 6 | 1 |
| 2ac2b67519965d88a0b2f3e87d05bd95 | 6 | 1 |
| 32cbedc5a8addbcb72cecaede6628a27 | 6 | 1 |
| 336eb65e4eddfa213bdca7b7553d1b7d | 6 | 1 |
| 3cbfdb9e16a863844f801d2f6f85b9d4 | 6 | 1 |
| 3fd371a64e0dfb71191e88778e399166 | 6 | 1 |
| 4012a825b6c2a9d5f7f000ffa2f3b804 | 6 | 1 |
| 4039288326c4c33d230595096d7f7a8c | 6 | 1 |
| 413fd512711d026dbb57c61d50b5b99d | 6 | 1 |
| 4c1bfa5acbb4f5ba6294e6d315221b70 | 6 | 1 |
| 51f7204a5526878fce1e9b0a665c7313 | 6 | 1 |
| 57445fefe215e062cf14e152a9b046c7 | 6 | 1 |
| 5898d1884ceb9da6e9449897c5848f80 | 6 | 1 |
| 58fff748362d049c0b949fbe634dbcf0 | 6 | 1 |
| 5dd5659d3d254a31b10c2e2c80c94257 | 6 | 1 |
| 63055ff6b9eddd51c5ad49bfb87e040f | 6 | 1 |
| 68c182fc655acb3ddcd3908d94883d90 | 6 | 1 |
| 6e38983ff0c382995817f32ad4df0645 | 6 | 1 |
| 748a25fd2164b498dedae6660a67e1b8 | 6 | 1 |
| 7a8afdddd651c6be9c76d2a61be55210 | 6 | 1 |
| 813b6fe1367321f6c12a404fc6b4a1e5 | 6 | 1 |
| 87ee17afe2afb5453591ea9945a3d9b4 | 6 | 1 |
| 8aa5b24e9250134a79492f404c331e8b | 6 | 1 |
| 90723464194e2d958721a8923cd3ce58 | 6 | 1 |
| 91d4f062c177de4f6a8a56036eefd949 | 6 | 1 |
| 92aa893aff80af3153371d507523d654 | 6 | 1 |
| 94ff2a18dd12915a2964e8a32118ac55 | 6 | 1 |
| 9f093506932cfa0719b74a7bc9ecb7de | 6 | 1 |
| a3313c35895e63ca02287dcc3f2d83dc | 6 | 1 |
| a5b08b40f220c6e6846e06414866a017 | 6 | 1 |
| a872a747abdfed704ad21b1a61d87c4b | 6 | 1 |
| ab10045056f72913c38c99fb1df375b2 | 6 | 1 |
| b0573b11d906dec1ff40adab5433e110 | 6 | 1 |
| b165b77015517ae83eca8824bfe4941b | 6 | 1 |
| b94fe34b6ce2c740bc6bb24f2c6521fe | 6 | 1 |
| bd5a00f279b819ce0a45a11be18529f5 | 6 | 1 |
| c2075e8692da3efa3847972513c75763 | 6 | 1 |
| c8558c98a5b5f2bdc66a91f28df172d3 | 6 | 1 |
| df764ee8dd6d418231b294daca0b91a4 | 6 | 1 |
| e02cf77d3d1fb3710257805f08d2eaef | 6 | 1 |
| e235f993d6e31c54daffe32c2f4080da | 6 | 1 |
| e8d6e97b29098aa79d4b42eeb6f570d1 | 6 | 1 |
| f00d3c73db6a5446543a5a030c9ee666 | 6 | 1 |
| f3f94b9c8cb1f93fa45126decacff988 | 6 | 1 |
| f90040d15b2f636a1588d620d760daf2 | 6 | 1 |
| fc641db9b6bce5dcb31ecd8b9f9ab9b0 | 6 | 1 |
| fe4ef75ad52df4debf0a762f455da818 | 6 | 1 |
| ff22f757b3b820a665e4016596072044 | 6 | 1 |
| 04b42d8346d5ad717a1938b58f4de449 | 5 | 1 |
| 08dc388ec3a742a0f281534a673adcff | 5 | 1 |
| 092d2fb0671c1488e52ba9f030522dbc | 5 | 1 |
| 1290ad3a66ae7f7be8bd7da905191468 | 5 | 1 |
| 16b539742d21e35df8b682e4adb5c83f | 5 | 1 |
| 16cbed879c2c06c366b375a6dde16efa | 5 | 1 |
| 2211281cb86d9e48feea64d3dfc2e820 | 5 | 1 |
| 27423d2fae31945499aa731ea7945401 | 5 | 1 |
| 2813421209fd0ab2271037ee13369163 | 5 | 1 |
| 2b5f2bda2d8bd5309d78f6f13f82a382 | 5 | 1 |
| 2e8b27e98e69332fbbcc25e85d140e66 | 5 | 1 |
| 344ec4d31ea600fe301686dd50e46b36 | 5 | 1 |
| 3928ff3b8b706b84422318faca9a6bf6 | 5 | 1 |
| 3be9cb83a205c7156124d503370f5034 | 5 | 1 |
| 470e7cec85de9f79452c2b5c618e8eb3 | 5 | 1 |
| 54d61d17d70fb5a72c4ab5d6300bf690 | 5 | 1 |
| 575dc2b765b20e254108cbf808369fc4 | 5 | 1 |
| 5877ad001d5863e7f49cb30ae32c64e0 | 5 | 1 |
| 5894a3731af6eacdf1c44deb582d8acb | 5 | 1 |
| 665176aa92b9fc98499ab419d10f08b1 | 5 | 1 |
| 67c0ae8f94cf03d1febe7eb2fe2a1437 | 5 | 1 |
| 6b8d3f965295ac517322e2581eac0ecc | 5 | 1 |
| 6bfe12989ed86343bf75ebc5009a3be9 | 5 | 1 |
| 6eed5d691dbba4a351eb423585387a2f | 5 | 1 |
| 6f9833c79bf4a9a94c80123bb10fb284 | 5 | 1 |
| 709ce6ef7cbe3a2438c55de7a4e3eb5d | 5 | 1 |
| 742ba4b4e5e79eeaaabc4a4a4dfb5ad0 | 5 | 1 |
| 8d8c0c7d7bced082ed23b0c87d7818cb | 5 | 1 |
| 8dd667e93828086966a84feef96f8abf | 5 | 1 |
| 8de5c189eaafff7c78da4287d5618e87 | 5 | 1 |
| 9218e1c6eb287ff2148d268ab5848122 | 5 | 1 |
| 9287bef048a94247af6ab506615a89f0 | 5 | 1 |

**Table S4.** Feature details in each sample.

|  | **sequence counts** |
| --- | --- |
| Minimum | 94467 |
| Median | 425849.0 |
| Mean | 429613.037037037 |
| Maximum | 871373 |
| Total | 11599552 |

**Table S5.** Demultiplexed sequence counts summary.

| **sample ID** | **forward sequence count** | **reverse sequence count** |
| --- | --- | --- |
| SRR13685040 | 307370 | 307370 |
| SRR13685041 | 127861 | 127861 |
| SRR13685042 | 339430 | 339430 |
| SRR13685043 | 553152 | 553152 |
| SRR13685044 | 342267 | 342267 |
| SRR13685045 | 501827 | 501827 |
| SRR13685046 | 723085 | 723085 |
| SRR13685047 | 317470 | 317470 |
| SRR13685048 | 94467 | 94467 |
| SRR13685049 | 230718 | 230718 |
| SRR13685050 | 543346 | 543346 |
| SRR13685051 | 185164 | 185164 |
| SRR13685052 | 425849 | 425849 |
| SRR13685053 | 697249 | 697249 |
| SRR13685054 | 449394 | 449394 |
| SRR13685055 | 582692 | 582692 |
| SRR13685056 | 255673 | 255673 |
| SRR13685057 | 761162 | 761162 |
| SRR13685058 | 783018 | 783018 |
| SRR13685059 | 275227 | 275227 |
| SRR13685060 | 497327 | 497327 |
| SRR13685061 | 871373 | 871373 |
| SRR13685062 | 498568 | 498568 |
| SRR13685063 | 158935 | 158935 |
| SRR13685064 | 197627 | 197627 |
| SRR13685065 | 565409 | 565409 |
| SRR13685066 | 313892 | 313892 |

**Table S6.** Sequence counts in per sample.

| **Forward Reads** | |  | **Reverse Reads** | |
| --- | --- | --- | --- | --- |
| **Total Sequences Sampled** | **10000.0** |  | **Total Sequences Sampled** | **10000.0** |
| 2 % | 300 nts |  | 2 % | 300 nts |
| 9 % | 300 nts |  | 9 % | 300 nts |
| 25 % | 300 nts |  | 25 % | 300 nts |
| 50 % (Median) | 300 nts |  | 50 % (Median) | 300 nts |
| 75 % | 300 nts |  | 75 % | 300 nts |
| 91 % | 300 nts |  | 91 % | 300 nts |
| 98 % | 300 nts |  | 98 % | 300 nts |

**Table S7.** Demultiplexed sequence length summary.

| **Metric** | **Sample** |
| --- | --- |
| Number of sample | 25 |
| Number of features | 650 |
| Total frequency | 22851 |
| **Table S8.** Table summary after filtering | |
| **Metric** | **Frequency** |
| Minimum frequency | 2 |
| 1st quartile | 192 |
| Median frequency | 621 |
| 3rd quartile | 1066 |
| Maximum frequency | 5166 |
| Mean frequency | 914 |

**Table S9.** Frequency per sample after filtering

| **Metric** | **Metadata** | **H** | **p-value** | **Significance** |
| --- | --- | --- | --- | --- |
| Observed ASVs | Sex | 0.32 | 0.5716 | n.s. |
| Observed ASVs | Tissue | 1.46 | 0.6921 | n.s. |
| Shannon index | Sex | 0.32 | 0.5716 | n.s. |
| Shannon index | Tissue | 1.68 | 0.6422 | n.s. |

Table S10. Kruskal–Wallis results for alpha diversity

| Bacteria family | Bacteria family | rho | p_value |
| --- | --- | --- | --- |
| *Enterococcaceae* | *Streptococcaceae* | -0.23673 | 0.25456 |
| *Staphylococcaceae* | *Streptococcaceae* | -0.21733 | 0.296684 |
| *Pasteurellaceae* | *Streptococcaceae* | -0.21714 | 0.297114 |
| *Enterococcaceae* | *Staphylococcaceae* | -0.18126 | 0.385867 |
| *Pasteurellaceae* | *Staphylococcaceae* | -0.08688 | 0.679645 |
| *Enterococcaceae* | *Pasteurellaceae* | 0.079687 | 0.704955 |

**Table S10.** The correlations between identified families based on Spearman's correlation.

| Bacteria genus | Bacteria genus | rho | p_value |
| --- | --- | --- | --- |
| *Enterococcus* | *Streptococcus* | -0.24727 | 0.233381 |
| *Staphylococcus* | *Streptococcus* | -0.21733 | 0.296684 |
| *Enterococcus* | *Staphylococcus* | -0.18183 | 0.384371 |

**Table S11.** The correlations between identified genera based on Spearman's correlation.

## Supplementary Figures


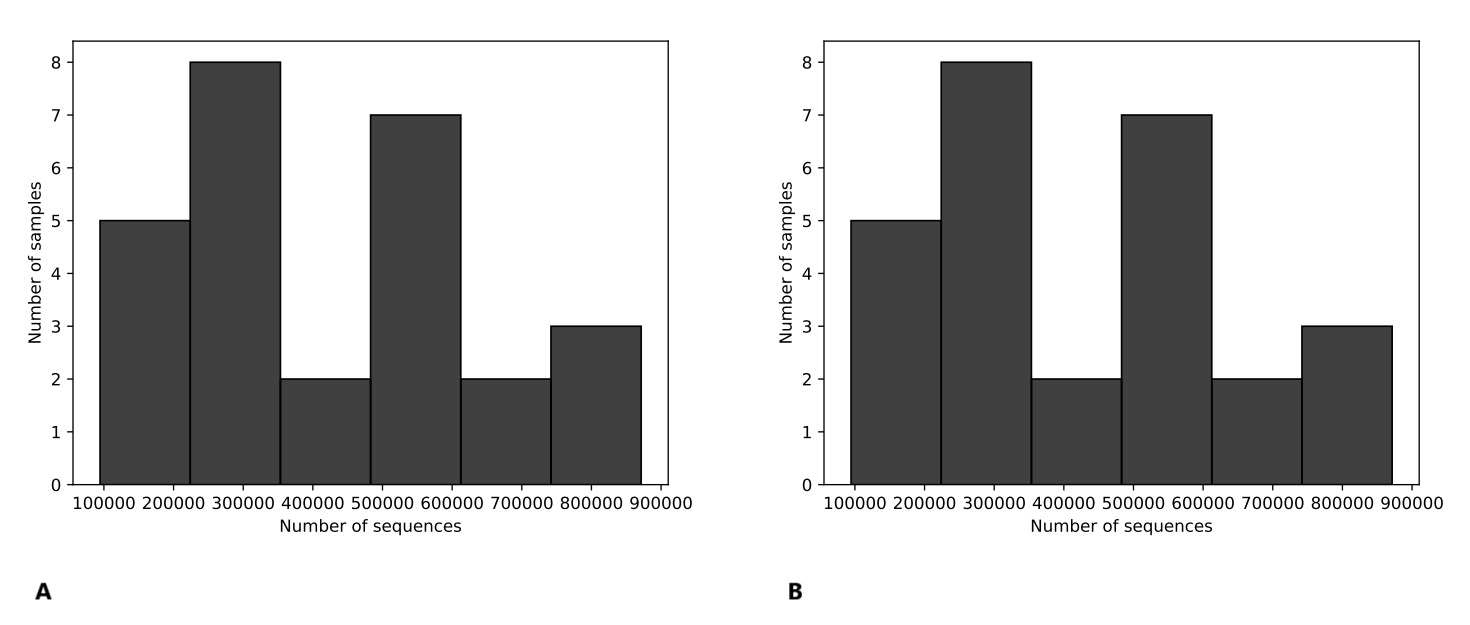


**Figure S1.** Demultiplexed sequence counts summary plot (A) Forward Reads Frequency, (B) Reverse Reads Frequency.


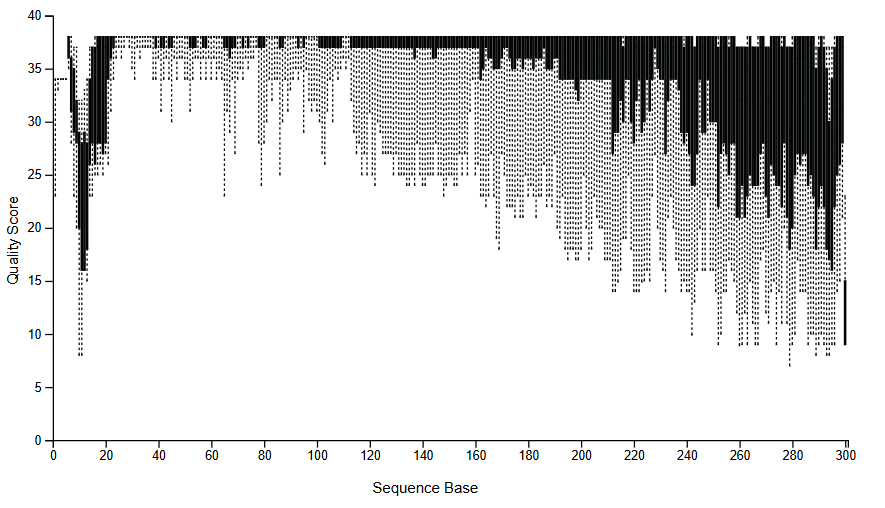


**Figure S2.** Quality plot for forward reads (These plots were generated using a random sampling of 10000 out of 11599552 sequences without replacement. The minimum sequence length identified during subsampling was 300 bases. Outlier quality scores are not shown in box plots for clarity.)


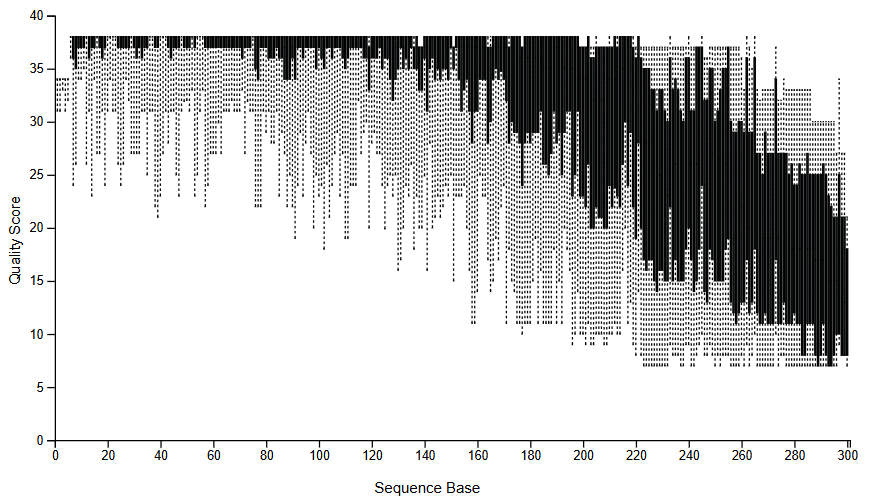


**Figure S3.** Quality plot for reverse reads (The plot at position 131 was generated using a random sampling of 10000 out of 11599552 sequences without replacement. The minimum sequence length identified during subsampling was 300 bases. Outlier quality scores are not shown in box plots for clarity).
